# Recent Advances in One-Pot Modular Synthesis of 2-Quinolones

**DOI:** 10.3390/molecules25225450

**Published:** 2020-11-20

**Authors:** Wan Pyo Hong, Inji Shin, Hee Nam Lim

**Affiliations:** 1 School of Advanced Materials and Chemical Engineering, Daegu Catholic University, 13-13, Hayang-ro, Hayang-eup, Gyeongsan-si, Gyeongbuk 38430, Korea; wphongw@cu.ac.kr; 2 Department of Fine Chemistry, Seoul National University of Science and Technology, 232 Gongneung-ro, Nowon-gu, Seoul 01811, Korea; 3 Eco-Friendly New Materials Research Center, Therapeutics&Biotechnology Division, 141, Gajeong-ro, Yuseong-gu, Daejeon 34114, Korea

**Keywords:** 2-quinolone, one-pot, catalysis, drug, multi-component, cascade, *N*-heterocycle

## Abstract

It is known that 2-quinolones are broadly applicable chemical structures in medicinal and agrochemical research as well as various functional materials. A number of current publications about their synthesis and their applications emphasize the importance of these small molecules. The early synthetic chemistry originated from the same principle of the classical Friedländer and Knorr procedures for the preparation of quinolines. The analogous processes were developed by applying new synthetic tools such as novel catalysts, the microwave irradiation method, etc., whereas recent innovations in new bond forming reactions have allowed for novel strategies to construct the core structures of 2-quinolones beyond the bond disconnections based on two classical reactions. Over the last few decades, some reviews on structure-based, catalyst-based, and bioactivity-based studies have been released. In this focused review, we extensively surveyed recent examples of one-pot reactions, particularly in view of modular approaches. Thus, the contents are categorized as three major sections (two-, three-, and four-component reactions) according to the number of reagents that ultimately compose atoms of the core structures of 2-quinolones. The collected synthetic methods are discussed from the perspectives of strategy, efficiency, selectivity, and reaction mechanism.

## 1. Introduction

**Figure 1:**
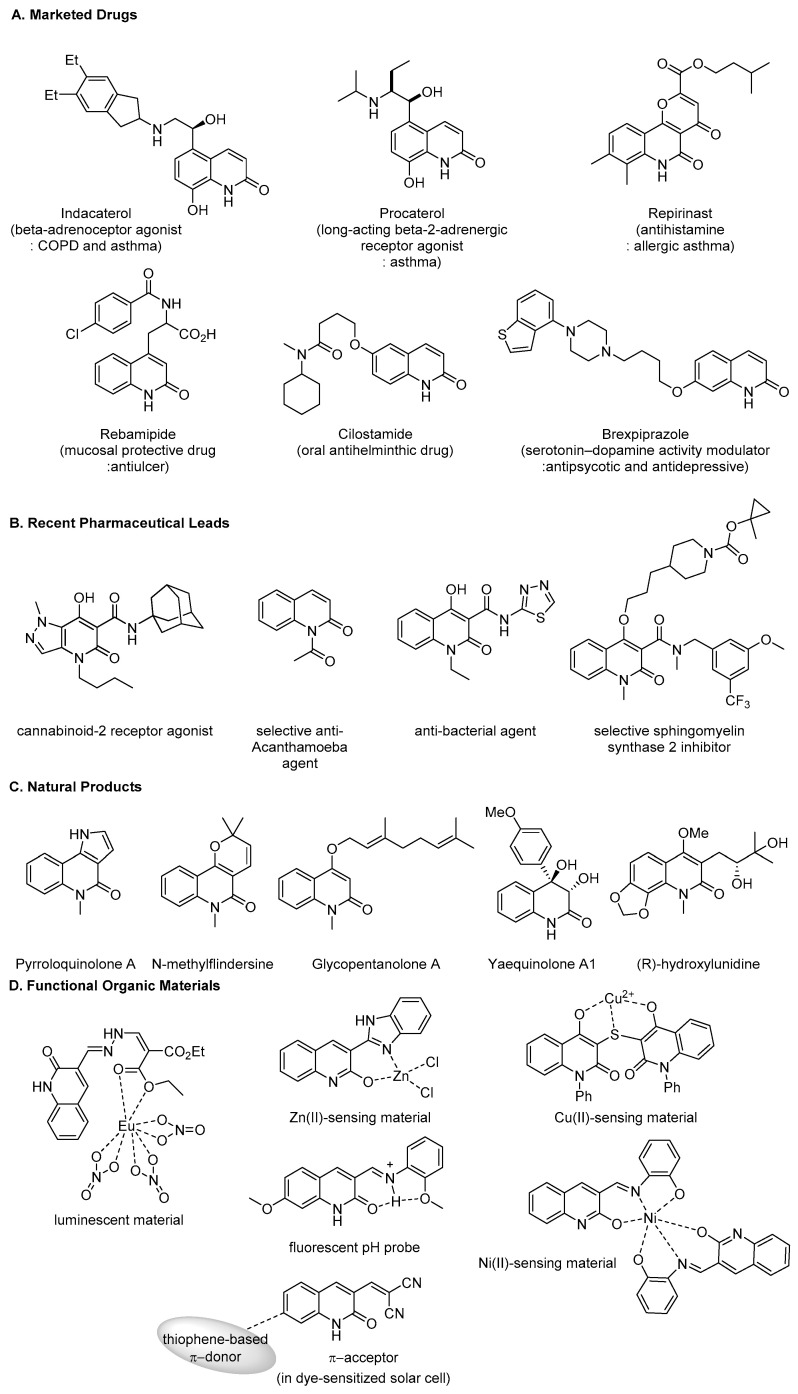
Recent examples of functional molecules containing 2-quinolone scaffold.

Quinolone and its structural congeners are versatile chemical entities that are ubiquitously present as core structures of pharmaceuticals, agrochemicals, and functional organic materials. Since the first identification of quinine from *Cinchona* bark in 1811, the isomeric compounds 2(1*H*)-quinolone and 4(1*H*)-quinolone have become representative twin molecules of pharmaceutical interest. In the pharmaceutical market, 4-quinolone drugs have attracted great attention since the discovery of nalidixic acid in 1962 [1]. Generations of several blockbuster fluoroquinolone antibiotics have been successfully commercialized. Moreover, 2-quinolones are a significant topic in the research area of medicinal chemistry as well. To date, six compounds in this class have been marketed (Figure 1A). For example, Indacaterol, Procaterol, and Repirinast are used for the treatment of respiratory diseases. Rebamipide is an antiulcer agent and Cilostamide is an antihelminthic drug. Brexipiprazole is a successor of the top-selling generic antipsychotic agent, Aripiprazole. Targets on this scaffold, β-adrenergic receptors [2,3,4,5], muscarinic acetylcholine receptors [6,7,8], vascular endothelial growth factor receptors [9,10], poly [ADP-ribose] polymerase-1 [11,12,13,14,15], tyrosine-protein kinases [16], and serine/threonine-protein kinases [17,18,19], have been extensively studied for decades. In addition to such target investigations, new lead compounds disclosed in recent reports [20,21,22,23,24] imply further potential pharmaceutical utility of the skeleton (Figure 1B).

In addition, 2-quinolones are embedded as core structures in natural products [25,26,27,28,29,30,31,32,33,34,35,36,37,38,39,40,41,42,43]. While a number of naturally occurring derivatives have been released, early reports revealed hybrid compounds that are derived from an isoprene unit and quinolones, namely hemiterpenoids [35,38,39,40,41,42,43]. Selected recent examples are presented in Figure 1C. Besides biological applications, 2-quinoline scaffolds are found in functional organic materials (Figure 1D) [44,45,46,47,48,49]. Due to the intrinsic photoactive physical properties of the conjugated system and *N*,*O*-based ligating ability, 2-quinolones could be utilized as cationic metal–sensors [45,46,47], luminescent materials [44], and fluorescent small molecule pH probes [45]. Moreover, they show great potential as π-electron acceptors in dye-sensitized solar cells, after the construction of a relevant electron reservoir at the 3-position of 2-quinolones [49].

**Figure 2:**
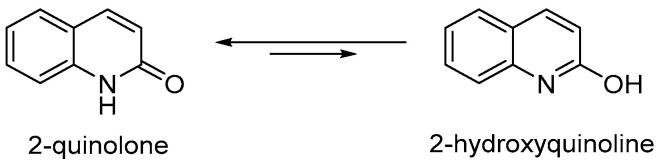
Tautomerization between 2-quinolone and 2-hydroxyquinolone.

Understanding the structural character of 2-quinolones is critical to the design of drug-like molecules with optimal binding affinity. The actual structure is deeply related to the external factors that influence tautomeric forms of 2-quinolones during the biological process [50,51,52,53]. Accordingly, studies on the tautomerism of 2-quinolone (Figure 2) have been of great interest in a similar manner as 4-quinolones [54,55,56,57,58,59]. The tautomerism has been studied by spectroscopic and computational methods [60,61,62,63,64]. The dominant presence of 2-quinolones over 2-hydroxyquinoline in nonaqueous phase or in solid state has been explained by the hydrogen-bonded dimeric stabilization of 2-quinolones [65]. In addition to hydrogen bonding, the equilibrium between the two forms appears to be determined by factors such as solvent polarity [66], steric hindrance, and the electronic properties of substituents [67]. Studies on the equilibrium of six-membered *N*-heterocycles indicates that the reduced aromaticity may contribute to the tautomerization of 2-quinolones as well [68].

**Figure 3:**
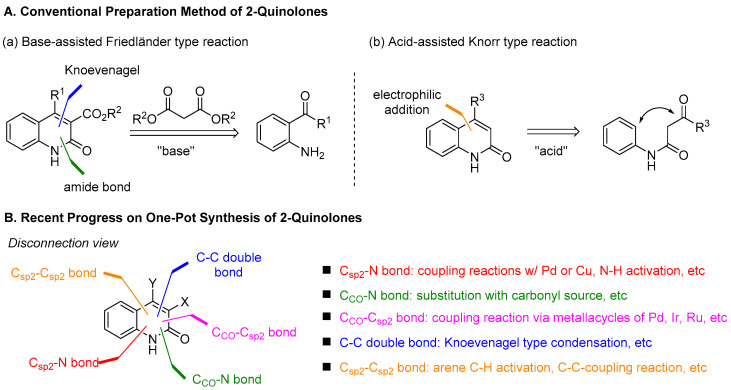
Overview of classical and recent approaches to 2-quinolones.

The development of synthetic methodologies for 2-quinolones has a long history. Conventional approaches represent two types of reaction: Friedänder-type reaction and Knorr-type reaction (Figure 3A). Holding high practicality and scalability for setting a good platform for further functionalization, the two methods have been used in a complementary fashion. The Friedänder-type reaction is the base-assisted intermolecular reaction between 2-aminoacylarenes and dialkyl malonates [69,70]. Considering bond disconnections, it needs a C–C double bond formation via Knoevenagel reaction and a C–N amide bond formation via nucleophilic substitution of amines to the esters. In the acid-assisted Knorr-type reaction [71,72,73,74,75,76,77], electrophilic aromatic substitution followed by dehydration forms C_sp2_–C_sp2_ bonds. While the two methods have inspired the development of numerous procedures based on the same bond disconnection, recent studies show remarkable advances in the synthesis of this small molecule by varying strategies that originate from new standpoints of bond disconnections (Figure 3B).

Several reviews on the synthetic routes to 2-quinolones have been published; an overview of syntheses and biological applications was recently released by Aly and Abdou [78,79]. These two papers focused on the scaffold of 4-hydroxy-2-quinolones. Yamamoto published a review article focused on the transition-metal-catalyzed synthesis of 3- and/or 4-aryl-2-quinolones [80]. Vessally covered contributions with reference to intramolecular cyclization reactions of *N*-arylpropiolamides via hydroarylation or electrophilic cyclization [81]. Nishiwaki reviewed synthetic methodologies for nitroquinolones and examples of elaborative synthetic application using them [82]. A comprehensive review by Silva covered palladium-catalyzed reactions for 2- and 4-quinolone synthesis, some of which are one-pot methods for the preparation of 2-quinolones [83]. This focused review aims to provide an extensive survey of recently developed one-pot intermolecular reactions to prepare functionalized 2-quinolone derivatives; it limits the scope to examples involving the construction of the core structures but not including mere functionalization of 2-quinolones. The contents are divided into three parts: (1) two-component reaction, (2) three-component reaction, and (3) miscellaneous reactions. The miscellaneous part includes four-component reactions and a set of novel approaches for the synthesis of more complex molecules or structural analogs that have heterocycles fused to the lactam nucleus.

## 2. Two-Component Reactions

### 2.1. Pd-Catalyzed Reactions

Over the past few decades, various strategies have been suggested to access 2-quinolones. Among these methods, palladium-catalyzed reactions using two coupling components have offered facile routes to 2-quinolone derivatives with a variety of substituents.

**Scheme 1:**
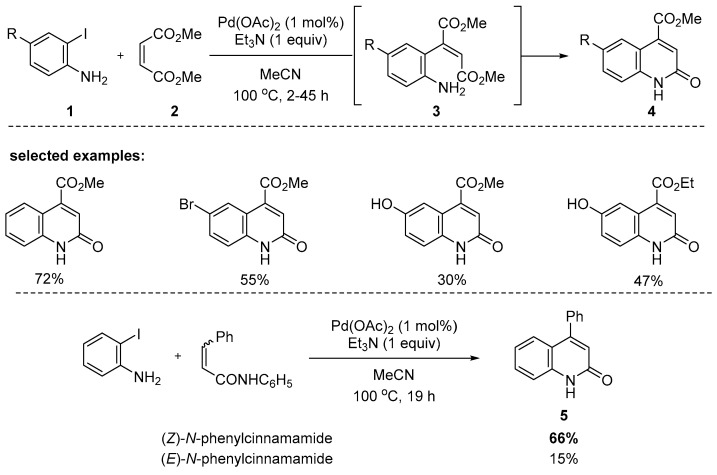
Synthesis of 2-quinolones by Pd-catalyzed reaction of 2-iodoanilines.

*o*-Iodoanilines have been largely exploited to access to 2-quinolone derivatives. Moreover, 4-Carbomethoxy-2-quinolones **4** could be efficiently synthesized from a dimethyl maleate and readily available 2-iodoanilines via the formation of (*Z*)-acrylic esters **3** in 30–72% yields (Scheme 1). The key intermediates **3** are formed by the Heck reaction. Moreover, 4-Phenyl-2-quinolone **5** could be also synthesized from 2-iodoaniline with (*Z*)-*N*-phenylcinnamamide in a 66% yield, whereas only in a 15% yield with the *E*-isomer. This indicates that (*Z*)-isomers are more suitable substrates for π-complexation with arylpalladium species than (*E*)-isomers [84].

**Scheme 2:**
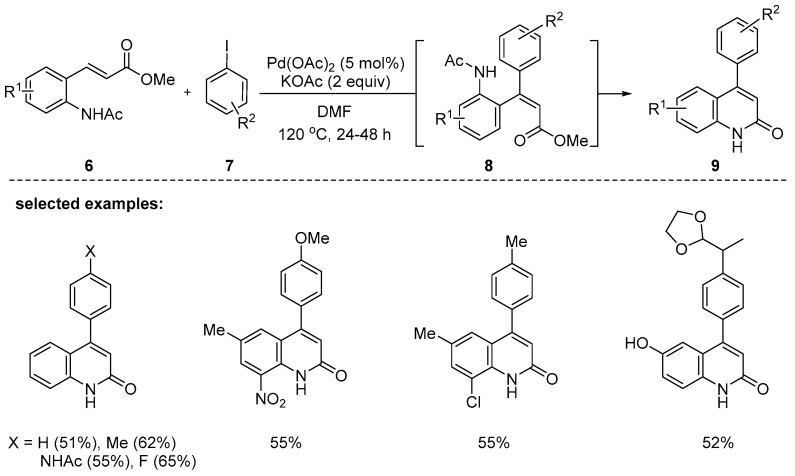
Synthesis of 4-aryl-2-quinolones through a sequential Heck reaction cyclization of methyl-*β*-(2-acetamidoaryl)acrylates **6** and aryl iodides **7**.

In 2006, Cacchi and coworkers reported the synthesis of 4-aryl-2-quinolones via intermediates **8** under Pd(OAc)_2_ and potassium acetate in *N,N*-dimethylformamide (DMF). The Heck-type coupling reaction between **6** and **7** smoothly proceeded to form intermediate **8**, which was isolated to identify the structure. Intermediate **8** underwent aminocyclization to provide the desired products **9** in 30–80% yields [85]. The Heck-type coupling reaction between **6** and **7** smoothly proceeded to form intermediates **8**; one of the intermediates **8** was isolated to identify the structure. The reaction tolerated electron-rich and electron-poor substituents in both methyl-*β*-(2-acetamidoaryl)acrylates **6** and aryl iodides **7** (Scheme 2).

**Scheme 3:**
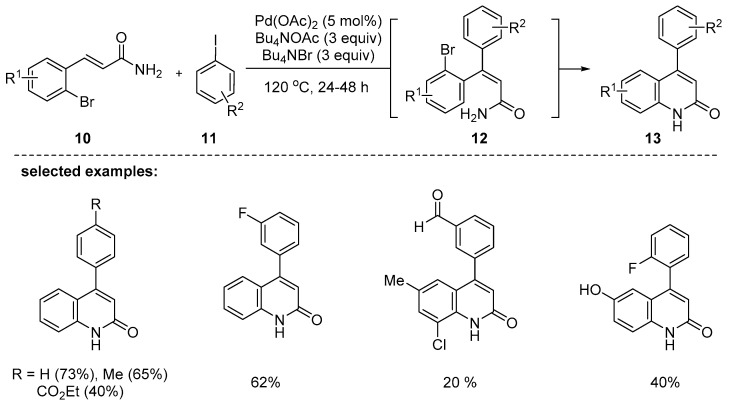
Synthesis of 4-aryl-2-quinolones through a sequential Heck reaction/amination process of β-(2-bromoaryl) acrylamides **10** with aryl iodides **11**.

In 2007, the same authors reported a phosphine ligand-free palladium-catalyzed domino Heck-cyclization reaction. As a complement to the previous work [85], acrylamides **10** instead of acrylic esters **6** were successfully utilized for the synthesis of 4-aryl-2-quinolones [86]. *β*-(2-Bromoaryl)acrylamides **10** reacted with aryl iodides **11** with electron-poor or -rich groups and the desired products **13** were obtained in 20–80% yields in the presence of molten Bu_4_NOAc and Bu_4_NBr combination (Scheme 3). A palladium-catalyzed pseudo-domino sequence was involved through Heck-type coupling followed by the Buchwald–Hartwig amination. It is noteworthy that phosphine ligand-free palladium-catalyzed domino Heck cyclization reaction is one of the few cases [87]. The reactive palladium nanoparticles stabilized by the molten Bu_4_NOAc and Bu_4_NBr salts mixture were tentatively suggested as the catalyst with high surface area.

**Scheme 4:**
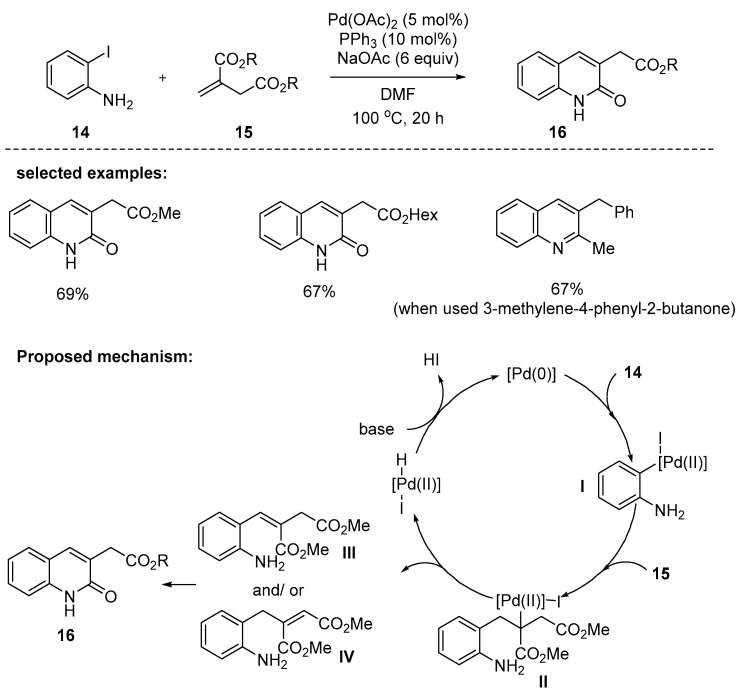
Synthesis of 3-substituted 2-quinolones through a sequential Heck reaction/cyclization process of 2-iodoaniline **14** with dialkyl itaconates **15**.

In 2007, Cho and Kim reported that the reaction between 2-iodoaniline and dialkyl itaconates furnished 2-quinolones possessing carboalkoxymethyl substituents at the C-3 position. The optimized conditions were found to involve Pd(OAc)_2_, PPh_3_, and NaOAc as a base at 100 °C for 20 h. Various dialkyl itaconates were investigated and the desired products were obtained in 67–76% yields [88]. Meanwhile, the reaction of *α*, *β*-unsaturated ketones possessing benzyl substituent at *α*-position, such as 3-methylene-4-phenyl-2-butanone, afforded a 2,3-dialkylsubstituted quinoline, not a quinolone. The reaction mechanism involves the oxidative addition of iodoaniline **14** to a catalytically active Pd(0) complex. Then, the arylpalladium species **I** adds across the alkene **15** to give alkylpalladium species **II**, followed by *β*-hydrogen elimination to afford the Heck products **III** and/or **IV**, which further cyclize to produce 2-quinolones **16** (Scheme 4).

**Scheme 5:**
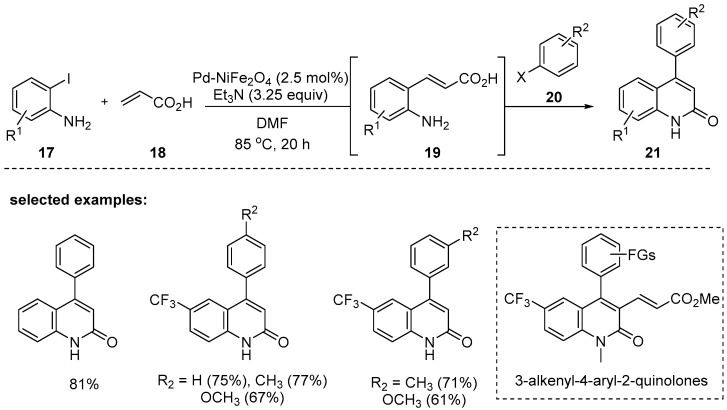
Pd/NiFe_2_O_4_-catalyzed one-pot synthesis of 4-aryl-2-quinolone derivatives.

In 2011, a unified strategy was reported by Borhade and Waghmode via two sequential Heck coupling reactions: (1) the first Heck-type Pd-NiFe_2_O_4_-catalyzed coupling reaction of *o*-iodoaniline **17** and acrylic acid **18** to afford the 2-aminocinnamic acid **19**, (2) another Heck coupling reaction of **19** and haloarenes **20** in the same pot to give **21** [89]. It turned out that the use of heterogeneous Pd-NiFe_2_O_4_ is a significant advantage in the separation process because it can be easily removed by applying an external magnetic field. Better yields with the aryl iodides were achieved compared with bromoarenes. The preparation of pharmaceutically important 3-alkenyl-4-aryl-2-quinolones by using the key intermediate **21** was demonstrated in this report (Scheme 5).

**Scheme 6:**
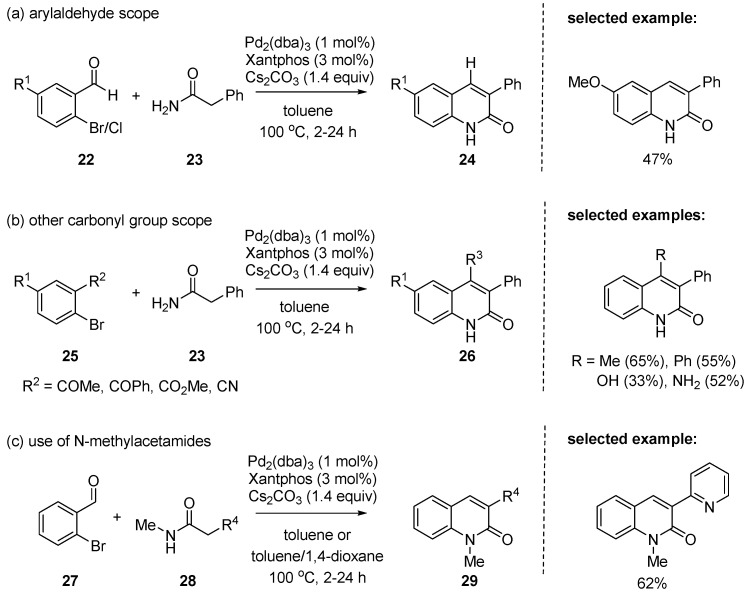
Synthesis of 2-quinolones by tandem Pd(0)-catalyzed amination/aldol condensation of aryl halides with acetamides.

In 2004, Manley reported that Pd-catalyzed annulation of *o*-halo-substituted benzaldehydes **22** with primary amides **23** gave 3-aryl-2-quinolones in moderate to excellent yields [90]. In the reaction, the C–C double bond was constructed by Knoevenagel condensation and the C–N cross-coupling was enabled by the Buchwald–Hartwig amination. The electronically varied substituents at the benzene ring were all compatible (Scheme 6a). Besides *o*-halo-substituted benzaldehydes, the substrates including 2-bromoacetophenone, 2-bromobenzophenone, methyl 2-bromobenzoate, methyl 2-bromo-5-phenylbenzoate, and 2-bromobenzonitrile worked well in the given reaction conditions, affording the corresponding 3,4-disubstituted 2-quinolones (Scheme 6b). In addition to primary amides, secondary amides, e.g., *N*-methyl-2-aryl acetamides **28**, were also suitable substrates in the reaction to give *N*-methyl-2-quinolones **29** (Scheme 6c). The broad reaction scope appears to be practically useful.

**Scheme 7:**
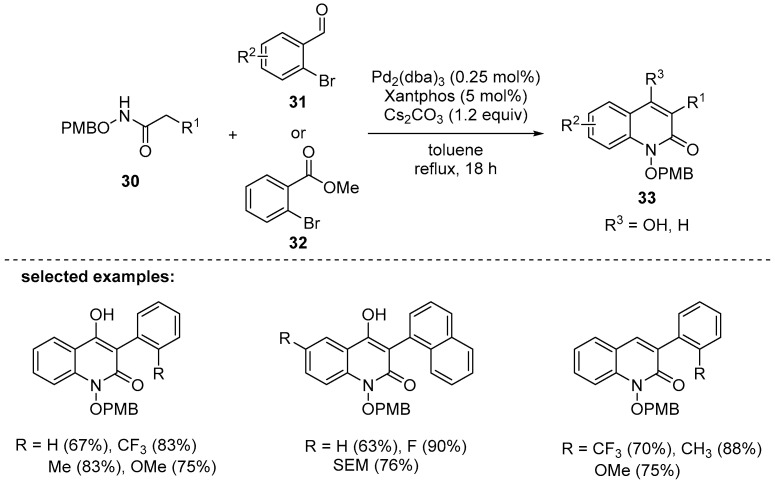
Synthesis of *N*-hydroxyquinolin-2(1*H*)-ones through a sequential Buchwald-type amidation/cyclization process with PMB (*p*-methoxybenzyl)-protected *N*-hydroxyamides.

Another Pd-catalyzed cascade reaction of Buchwald-type amidation and Knoevenagel reaction was reported in 2015. In the reaction, the substrates such as *N*-alkoxyamides **30** with benzaldehydes **31** or 2-bromo-methyl benzoates **32** were employed to yield the desired *N*-alkoxyquinolones in moderate to excellent yields, 63–96% (Scheme 7) [91]. With the optimized conditions (Pd_2_(dba)_3_, Xantphos, and Cs_2_CO_3_ in toluene), various *N*-alkoxyquinolin-2(1*H*)-ones **33** with a broad range of functional groups were successfully synthesized.

**Scheme 8:**
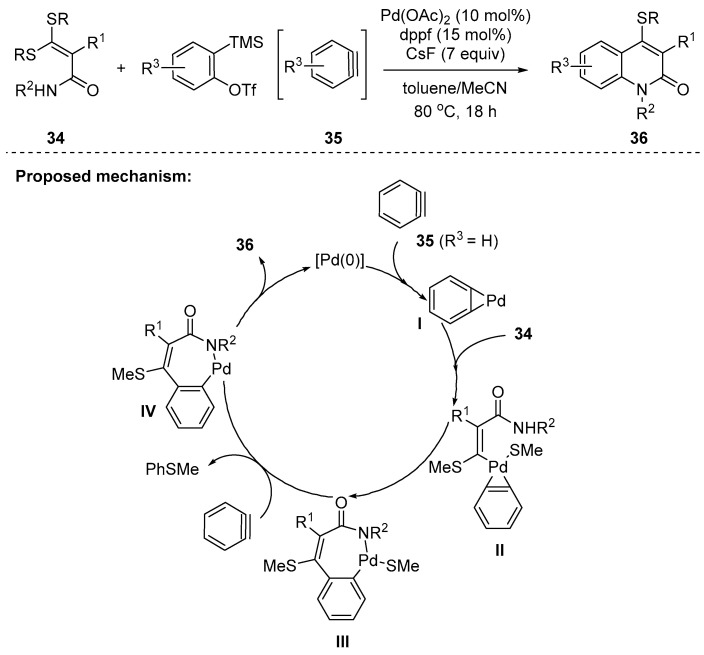
Synthesis of 2-quinolinones by Pd-catalyzed C–S activation/aryne insertion/intramolecular C–N coupling reaction.

In 2014, Liu and Wang developed a novel method for the synthesis of 4-alkylthio-substituted 2-quinolones **36**; C–S activation/aryne insertion/intramolecular C–N bond coupling reaction were three consecutive reactions under Pd-catalysis. The method used 2-carbamoyl ketene dithioacetals **34** and in situ generated arynes **35** (Scheme 8) [92]. Aryl- and alkyl substituents at the α-position of the ketene dithioacetals were tolerated; however, a ketene with a primary amide proved to be less efficient. The arynes with both electron-donating and -withdrawing groups were compatible in the optimized conditions to give 2-quinolones in moderate yields (41–51%). The mechanism of the annulation was proposed to be initiated by the cyclopalladation of an in situ generated aryne **35** that forms a palladacycle **I**. Then, insertion of **I** into the C–S bond of **34** occurs to afford the intermediate **III**, presumably via the species **II**. The subsequent desulfenylation followed by reductive elimination of **IV** would give 2-quinolones **36** and regenerated Pd(0) catalyst. The byproduct, PhSMe, was identified by high-resolution mass spectrometry.

**Scheme 9:**
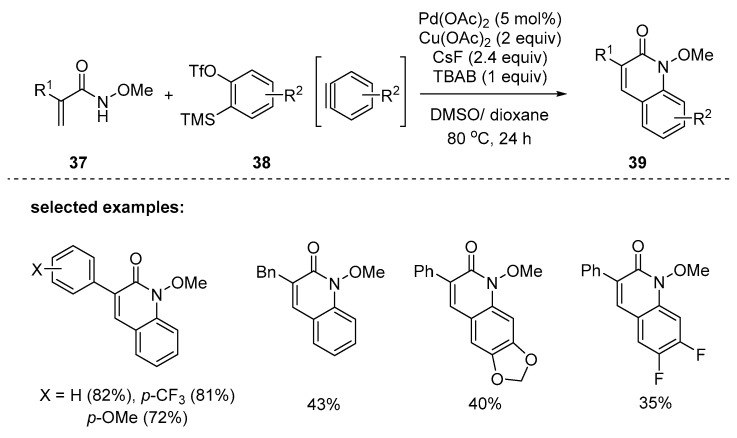
Synthesis of quinolones by Pd-catalyzed oxidative annulation between acrylamides and aryne precursors.

In 2015, Xu and coworkers disclosed a new methodology for the construction of *N*-methoxy 2-quinolones **39** by palladium-catalyzed N–H activation/Heck reaction [93]. The reaction coupled acryl amides **37** to arynes **38**, which was enabled by a catalytic Pd(OAc)_2_ and hyperstoichiometric use of Cu(OAc)_2_ and CsF in mixed solvents of dioxane and DMSO at 80 °C (Scheme 9). The acrylamides with electron-rich or -deficient aryl substituents at the α-position afforded **39** in good yields. However, the reactions using cinnamides or cryotonyl amides instead of acryl amides were not successful, thus indicating the importance of strain release. The control experiments and KIE (kinetic isotope effect) measurement showed that aminopalladation of **37** to benzyne is the key step of the catalytic cycle.

**Scheme 10:**
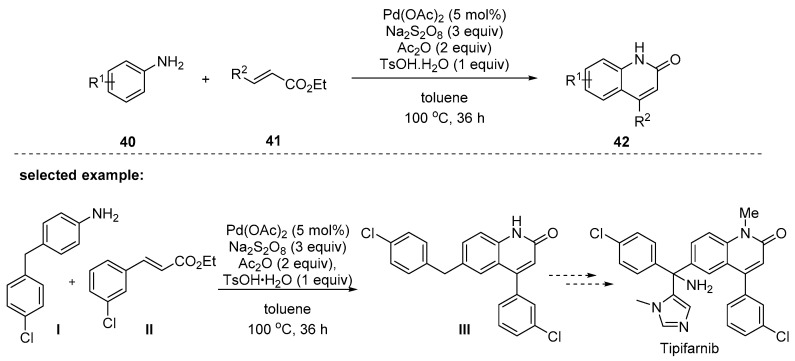
Synthesis of quinolin-2(1*H*)-ones by a Pd-catalyzed C–H activation/C–C bond formation/intramolecular cyclization reaction.

Liu and coworkers have reported a Pd-catalyzed cascade reaction of C–H bond activation/C–C bond formation/intramolecular cyclization by employing substituted anilines **40** and acrylates **41** [94]. During reaction optimizations, an in situ generated amide in the presence of Ac_2_O was the key to initiating arene C–H activation. The hydrolysis of the acetyl group was promoted by the addition of TsOH∙H_2_O (Scheme 10). The anilines **40** possessing electron-donating and -withdrawing groups worked smoothly to give the corresponding products **42** in moderate to excellent yields. However, the reactions using anilines possessing trifluoromethyl-, nitro-, or carbonyl groups at the *para* position were unsuccessful. Cinnamates **41** bearing electron-rich groups on R^2^ (aryl groups) showed higher reactivity than those with electron-deficient groups.

Quinolinone **III** was an important intermediate in the preparation of the potent, orally active inhibitor of farnesyl protein transferase, Tipifarnib. The developed method provided a more step-economical way to prepare it compared to the previous synthetic routes involving multi-step synthesis.

**Scheme 11:**
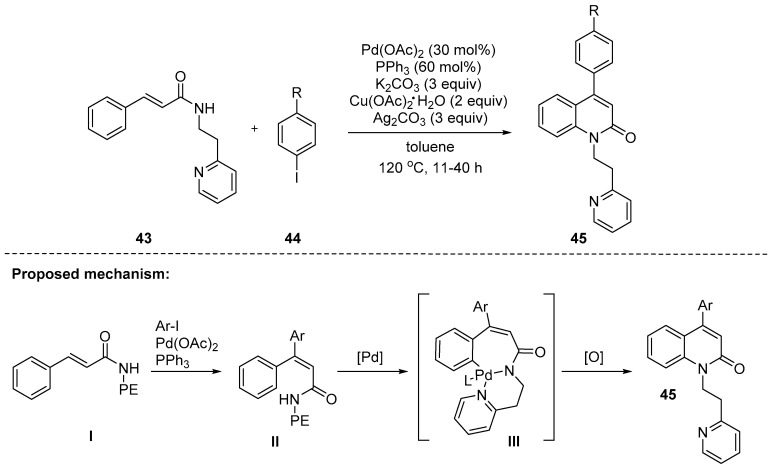
Synthesis of 4-aryl-2-quinolones through a sequential Heck reaction/intramolecular amidation process of cinnamides possessing 2-(pyridin-2-yl)ethanamine with aryl iodides.

In 2017, Hu and his coworkers disclosed a Pd(II)-catalyzed intermolecular Heck reaction/intramolecular C–H amidation reaction to prepare 4-aryl-2-quinolones **45** (Scheme 11). The reaction of cinnamides **43** containing 2-(pyridin-2-yl)ethanamine with aryl iodides **44** was carried out in the presence of Pd(OAc)_2_, PPh_3_, K_2_CO_3_, and copper/silver oxidants in toluene. The aryl iodides **44** with electron-donating groups such as methoxy and methyl groups efficiently worked to give the corresponding products in moderate yields. However, those with electron-withdrawing groups such as -F, -NO_2_, -CN, -CF_3_, and -CO_2_Me were less effective [95]. A proposed reaction mechanism is depicted in Scheme 11. The first step begins with the Heck reaction of **I** and aryl iodides and then an intramolecular C–H amidation reaction occurs to provide **45**. The C–H amidation was promoted by a pendant group of 2-(pyridin-2-yl)ethanamine which likely directs the Pd(II) catalyst towards the proximal C(*sp^2^*)–H bond to generate the palladacycle intermediate **III**.

### 2.2. Other Transition-Metal-Catalyzed Reactions Using Cu, Ag, Rh, Ir, and Ni

Decades of research have demonstrated that conventional palladium catalysts can be replaced by the first, second, and third row transition metals such as copper, silver, rhodium, and iridium for the preparation of 2-quinolones.

**Scheme 12:**
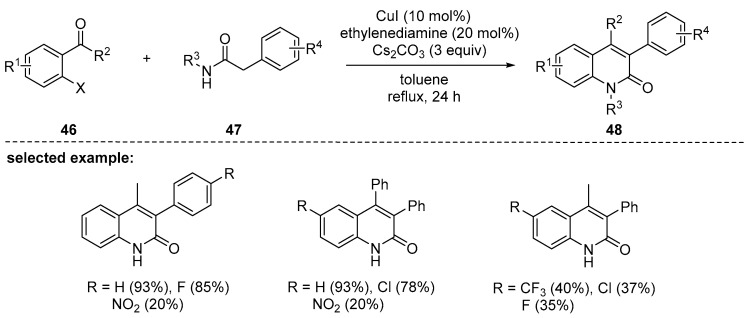
Copper iodide-catalyzed synthesis of 2-quinolones via cascade reactions of 2-halobenzocarbonyls with 2-arylacetamides.

The copper–ethylene diamine combination has been found to be an efficient catalytic system for the synthesis of 2-quinolinones **48** via cascade reactions of substituted 2-halobenzocarbonyls **46** with 2-arylacetamides **47** (Scheme 12). The use of ethylenediamine ligand has broadened the substrate scope. The reaction did not efficiently proceed in polar solvent such as *N*,*N*-dimethylformamide, whereas use of toluene ensured high conversion [96]. Although the protocol worked efficiently for less reactive aryl chloride, use of 2-iodobenzocarbonyls provided 2-quinolineone products **48** in better yields than 2-bromo- or 2-chlorobenzocarboyls in the given reaction conditions. Moreover, the reaction of arylacetamide **47** with electron-donating R^4^ substituents provided the desired products in higher yields than the reaction with an electron-withdrawing group (NO_2_).

**Scheme 13:**
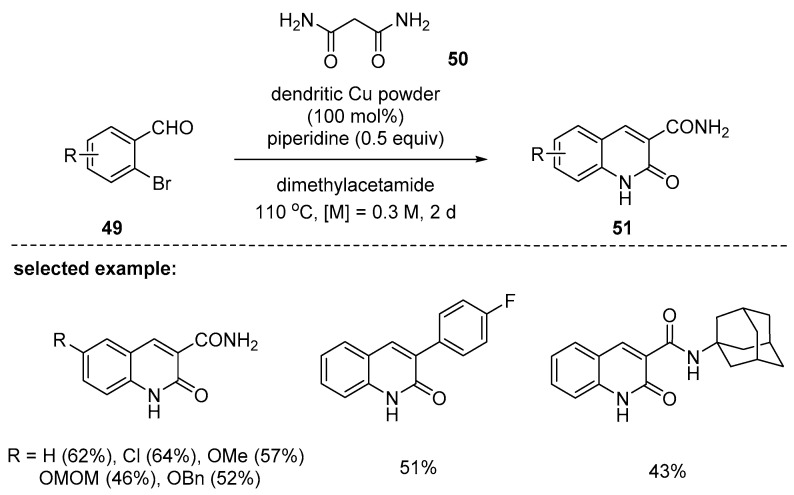
Preparation of 3-amido-2-quinolones using a dendritic Cu powder.

A contribution with Cu-catalyzed 2-quinolone synthesis was reported by Lim and coworkers in 2018. In their study, dendritic copper powder was discovered to promote both Knoevenagel condensation and C–N bond formation, and 3-amido-2-quinolones **51** were synthesized by the reaction with 2-bromobenzaldehydes **49** and malonamides **50** in one pot [97]. The key factor of the method was the use of an amine base that did not hydrolyze the amide. Besides the malonamides, other active methylenes containing *p*-fluorophenyl and bulky, adamantly substituted amides were found to be smoothly annulated to give the corresponding 3-substituted 2-quinolones (Scheme 13). During mechanistic studies, dendritic copper powder was proven to play a dual role as enabler of C–N bond formation and promoter of Knoevenagel condensation.

**Scheme 14:**
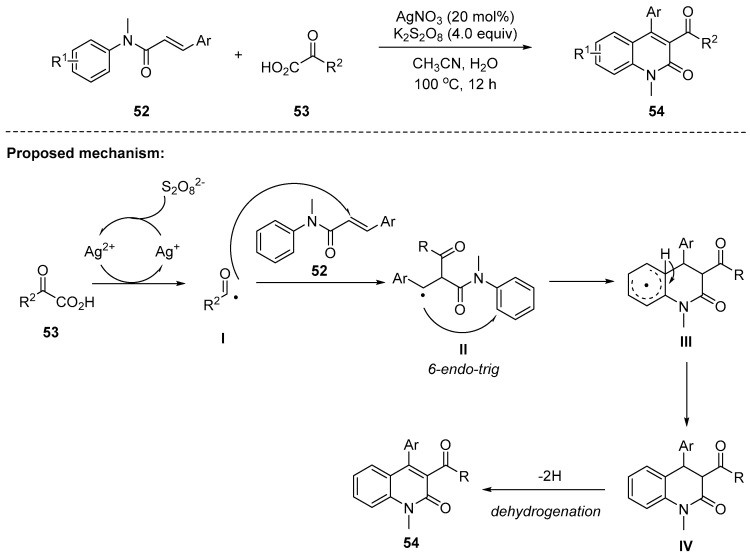
Radical cyclization toward substituted 2-quinolone derivatives.

Mai and coworkers have achieved the practical synthesis of 3-acyl-4-aryl-2-quinolones **54** using AgNO_3_ and K_2_S_2_O_8_ as a radical generator [98]. The reaction with higher loading (4 equiv) of oxidant K_2_S_2_O_8_ was better than that with lower loading (1.0–2.5 equiv) and the product was obtained in an 83% yield. A wide range of both electron-withdrawing and -donating substituents such as *o*-Cl, *p*-Br, *p*-OMe, and 3, 4-diOMe on the phenyl ring of cinnamic acid **52** were compatible. In addition, 2-oxo-2-phenyl acetic acids **53** containing electron-withdrawing and -donating groups proceeded smoothly to produce **54** in good yields. The proposed reaction mechanism of this radical tandem cyclization involves acyl radical trapping, generated from carboxylate **53**, by the α-position of the *N*-methyl-*N*-phenyl cinnamamides **52** followed by intramolecular cyclization to give an intermediate **III**. The aromatizative dehydrogenation likely results in the formation of the final products **54** (Scheme 14).

**Scheme 15:**
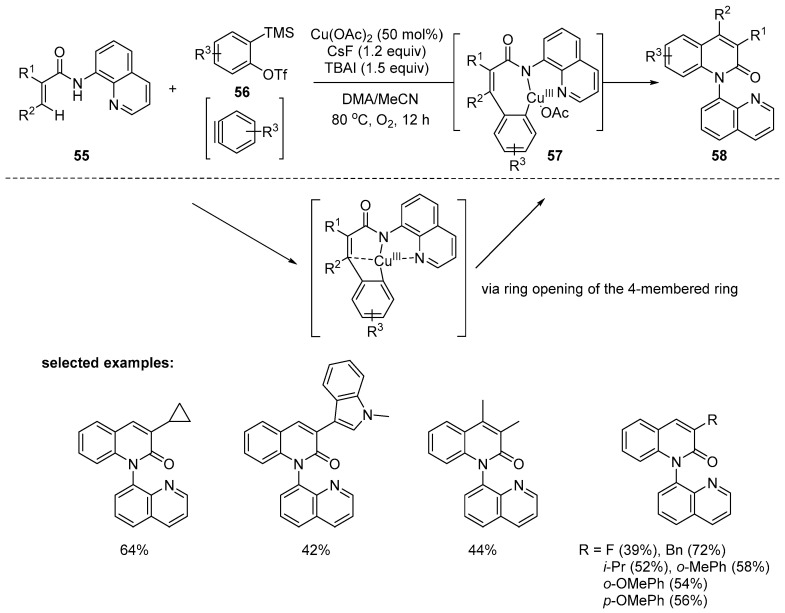
Synthesis of 2-quinolones via Cu-mediated C–H/N–H annulation.

Duane and Zhang reported copper-mediated one-pot cascade cyclization of acrylamides and arynes. A catalytic system of Cu(OAc)_2_ and O_2_ in DMA/MeCN was discovered for the efficient preparation of 4-aryl-2-quinolones **58**. It is notable that Cu(OAc)_2_ showed higher reactivity than Pd(OAc)_2_ [99]. A wide range of substituents on the α-position of monosubstituted acrylamides, e.g., aryl, alkyl, and cycloalkyl, were well tolerated to provide to 3-disubstituted 2-quinolones. However, only 39% yield was obtained with α-fluorinated acrylamide. In addition, α,β-disubstituted acrylamides could be utilized to generate 3,4-disubstituted 2-quinolinones in moderate yields. Through some control experiments, including an intermolecular KIE (value = 5.1) and radical trapping experiment, the single-electron transfer (SET) mechanism could be excluded. On the basis of experimental and computational studies, a plausible mechanism was proposed. The organometallic alkenyl C–H bond activation/addition to arynes via a Cu(III) intermediate **57**, presumably through the ring opening of the four-membered ring **I**, affords the expected products **58** with a new C–N bond after reductive elimination (Scheme 15).

**Scheme 16:**
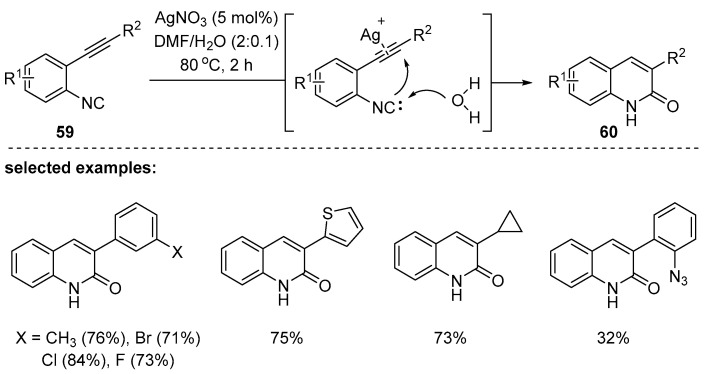
Synthesis of 3-aryl-2-quinolones by alkyne activation with Ag(I) catalyst.

In 2018, Kuhakarn and coworkers disclosed a novel annulation technique using *o*-alkynylisonitrile and H_2_O in the presence of a catalytic amount of silver nitrate [100]. While not clearly demonstrated, the proposed mechanism suggests that alkyne activation by a silver cation would be followed by nucleophilic addition of the carbanion to the activated alkynes; the carbanion seems to be generated by H_2_O addition to isocyanate (Scheme 16). Besides silver (I) salts, other metal complexes such as Cu(I), Cu(II), Zn(II), and Fe(III) as well as tosylic acid worked in the designed reaction system. A variety of 3-substituted 2-quinolone derivatives were successfully synthesized by the method.

**Scheme 17:**
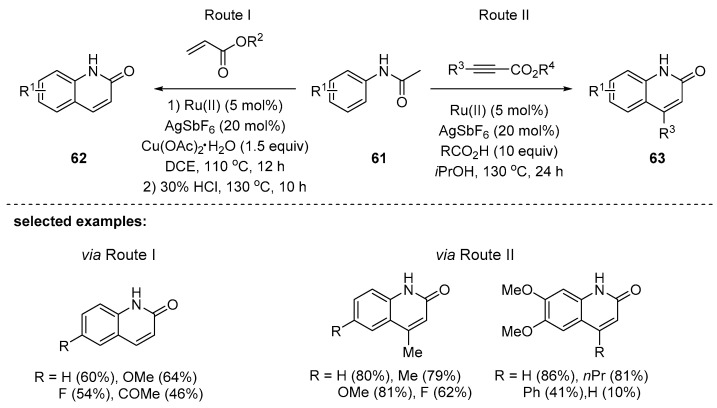
Ru-catalyzed annulation of anilides with acrylates or propiolates.

In 2014, Jeganmohan’s research group reported ruthenium-catalyzed synthesis of 2-quinolinones with anilides **61** and unsaturated hydrocarbons such as acrylates (Route I) or propiolates (Route II) [101]. The alkyl acrylates were first tested for the synthesis of 2-quinolones, and the reaction with methyl acrylate afforded the corresponding products in a better yield than those with ethyl or *n*-butyl acrylates. Anilides with various substituents such as OMe, F, or COMe were successfully reacted with methyl acrylates, providing the substituted 2-quinolones **62** in good yields (Route I). The substituted propiolates were also suitable substrates in the designed transformation. In this way, 4-substituted-2-quinolones **63** were obtained. It is interesting to see that each substrate optionally required a different type of acid source for better conversion, e.g., pivalic acid or acetic acid. The reaction scope was quite general; a variety of 2-quinolones with electron-donating, -withdrawing, or -neutral substituents were prepared in good to excellent yields (Scheme 17).

**Scheme 18:**
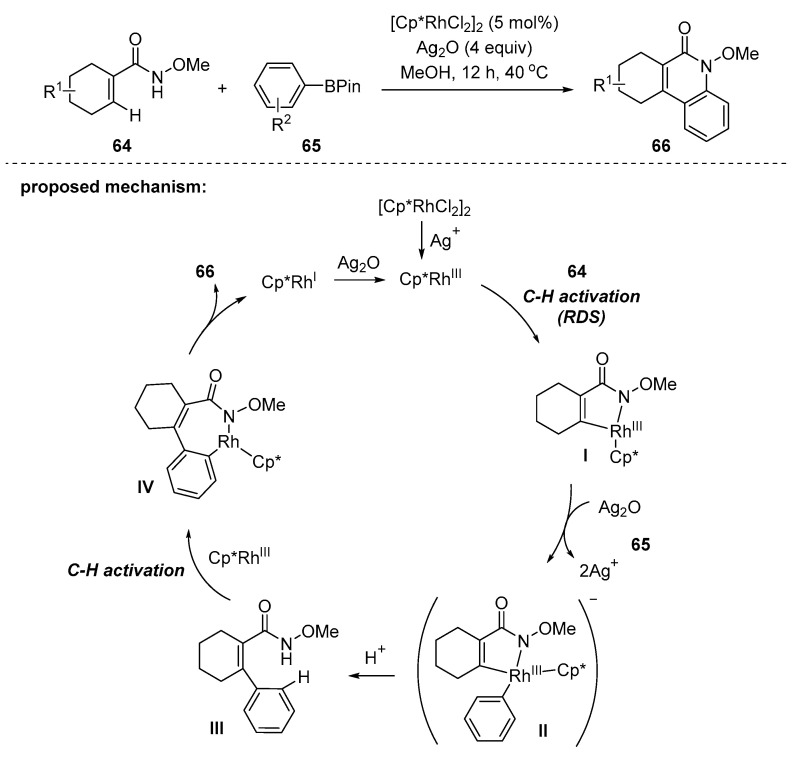
Rh-catalyzed synthesis of 3,4-cycloalkaquinolones using *N*-alkoxyamides and boronic esters.

In 2019, Zhu reported a Rh-catalyzed, amide-directed, double C–H bond activation strategy to prepare cycloalkaquinolones **66** with arylboronic acid **64** pinacol esters **65** (Scheme 18) [102]. The products were obtained in moderate to excellent yields and showed broad scope and high functional group tolerance. The mechanism firstly involves the formation of the silver-aided, five-membered rhodacycle **I** and transmetallation with boronic ester followed by reductive elimination, affording the *β*-C–H arylated product **III**. Then, the deprotonative coordination of the nitrogen in **III** to rhodium species triggers the second C–H bond activation, giving seven-membered rhodacycle **IV**. Finally, 2-quinolone **66** is provided after reductive elimination. The control experiments including H/D exchange and parallel KIE experiments strongly suggest that this reaction is initiated via the concerted metalation deprotonation (CMD) pathway, and the cleavage of the alkenyl C–H bond is probably the rate-determining step. This synthetic method successfully afforded cycloalkaquinolone scaffolds, which are often found in natural products with pharmacological interest.

**Scheme 19:**
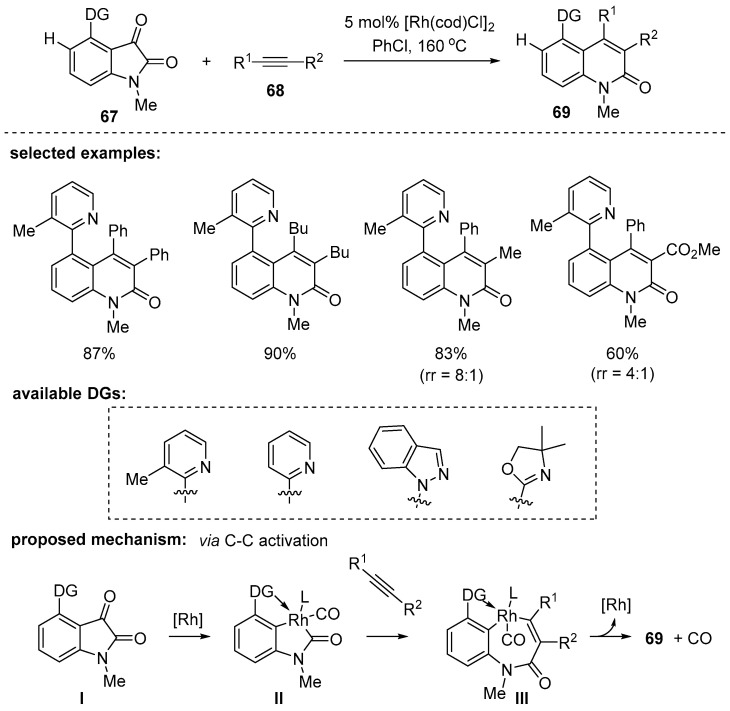
Rh-catalyzed decarbonylative coupling reaction.

Rong and Dong disclosed a unique two-component reaction between isatins **67** and alkynes **68** under rhodium-based catalytic conditions [103]. The reaction mode through a catalytic decarbonylative coupling reaction was a distinct point of the reaction design. Given the challenge of the selective C–C bond activation over arene C–H bond activation, the selective C–C bond activation was achieved by controlling the orientation of the directing group (Scheme 19). The methyl group at the 3-position of the pyridine ring was the key to ensuring the correct orientation which directs the rhodium complex to the ketone group of the isatins. During the mechanistic studies to prove the pathway by C–C bond activation, a Rh complex **II** (L = pyridine) was successfully isolated and, interestingly, it was generated at room temperature. However, alkyne insertion to **II** did not occur below 130 °C. This tentatively suggests that a high reaction temperature is required for either the following alkyne insertion step or reductive elimination step.

**Scheme 20:**
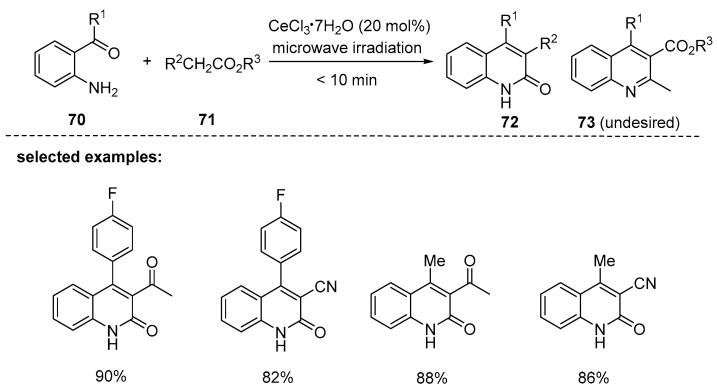
Microwave-assisted, solvent-free synthesis of substituted 2-quinolones by reaction of *o*-aminoarylketones with esters.

Wang and coworkers have developed a microwave-assisted, Friedländer-type condensation method using *o*-aminoarylketones and esters with a reactive α-methylene moiety (Scheme 20). Cerium chloride heptahydrate (CeCl_3_∙7H_2_O) was used as the catalyst and this common Lewis acid offered excellent yields, with high selectivity towards to 2-quinolones in only 10 min [104]. The reactions between *o*-aminoarylketones **70** and keto esters were initially nonselective, giving a mixture of 2-quinolones **72** and quinolines **73** under the copper catalysts (CuSO_4_∙5H_2_O, CuCl, and CuCl_2_∙2H_2_O). However, 2-quinolones **72** were obtained as major compounds after optimization in the presence of 0.2 equiv CeCl_3_∙7H_2_O under microwave irradiation (450 W).

**Scheme 21:**
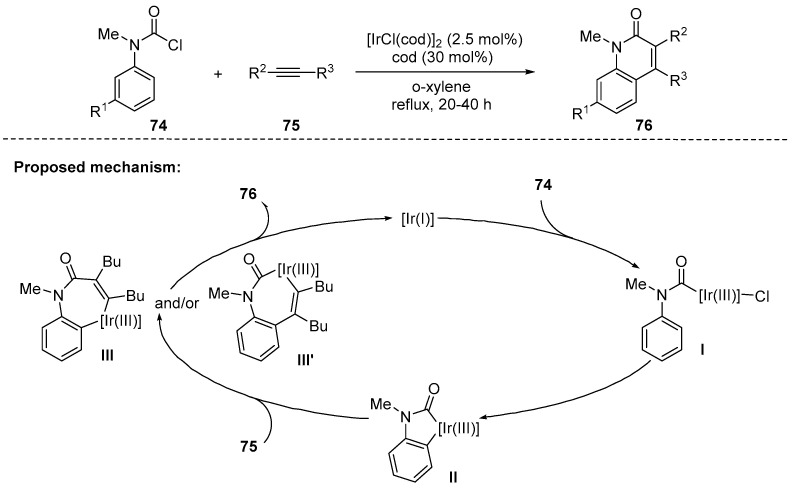
Ir-catalyzed cascade reaction using carbamoyl chlorides and internal alkynes.

In 2010, Tsuji and coworkers reported iridium-catalyzed synthesis of 3,4-disubstitued *N*-methyl-2-quinolones **76** using various *N*-methylarylcarbamoyl chlorides **74** and internal alkynes **75**. The catalytic conditions employed [IrCl(cod)]_2_ (cod = 1,5-cyclooctadiene) and cod as a dummy ligand. The reaction tolerated a wide range of substituents in both coupling partners. For example, symmetrical alkynes were well suited in this annulation, providing the desired products in good yields (62–67%). The selectivity was not practically high in cases of the unsymmetrical alkynes. However, it is notable that unsymmetrical alkynes possessing an ether functionality increased the regioselectivity, presumably by directing the effect of the oxygen atom [105]. A reaction pathway was proposed based on control experiments and literature precedents. Initially, Ir(I) is oxidized to Ir(III) by **74**, and **I** is then converted to five-membered iridacycle **II** by an arene sp^2^ C–H activation step. The formation of the iridacycle **II** plays a crucial role in suppressing the decarbonylation that can lead to deconstructive pathways. The intermediate **II** was firmly identified by X-ray crystallography of a phosphine-bound iridium phosphine complex. Then, the insertion of alkynes **75** yields seven-membered iridacycle **III** or **III′**. The subsequent reductive elimination affords 2-quinolones **76** by regenerating the Ir(I) catalyst (Scheme 21).

**Scheme 22:**
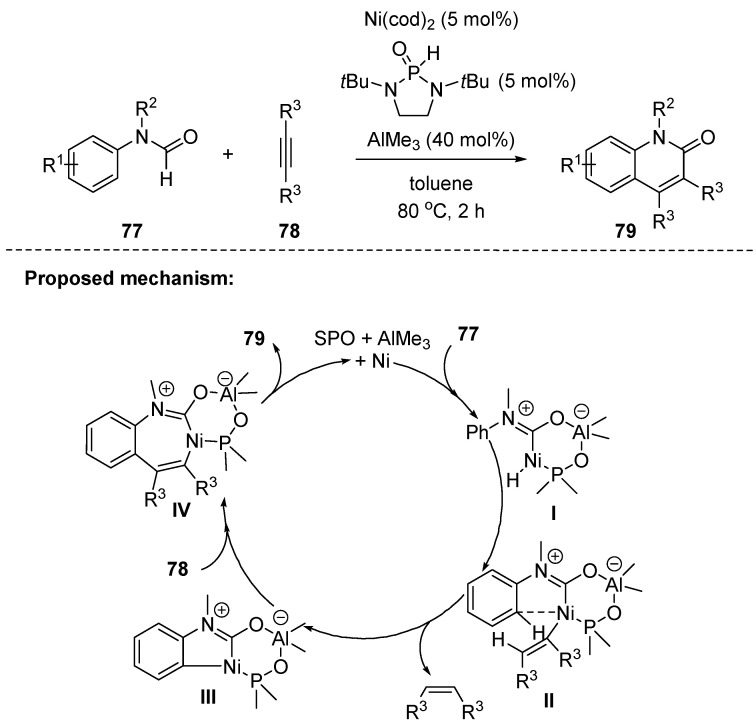
Ni–Al cooperative bimetallic catalysis with arylformamides and internal alkynes.

Recently, Ye and coworkers reported a new route to 2-quinolones using arylformamides **77** and alkynes **78** via a Ni–Al cooperative bimetallic catalysis. This novel strategy features nonchelated dual C–H activation, discovery of a novel secondary phosphine oxide (SPO) ligand in bimetallic catalysis, wide substrate scope, and good functional group tolerance [106]. The regioselectivity was highly dependent on substituents of alkynes. For example, the reaction of asymmetric dialkyl acetylenes resulted in poor regioselectivities, while the use of alkylphenyl acetylenes led to good regioselectivities (>10:1). It is suggested that the regioselectivity is determined by the electronic factors of the substituents at both sides of alkynes. In cases using alkylphenyl acetylenes, the phenyl was proximate to the carbonyl groups. This is due to favoring a more stable benzylic nickel species during the second alkylphenyl alkyne insertion to the nickelacycle **III**. The control experiments indicated that the SPO played the role of a bifunctional ligand to facilitate the reaction by the unique coordination mode, as shown in proposed mechanism in Scheme 22. A plausible mechanism for this process involves formation of the intermediate **I** via oxidative addition of Ni(0) to the formyl C–H bond. This step is proposed to be likely assisted by the SPO-bound Ni–Al complex, which is followed by Ni–H addition across the alkynes **75**. Next, the aryl C–H bond cleavage forms the nickelacycle **III**. The subsequent alkyne insertion and reductive elimination affords **76**.

### 2.3. Transition-Metal-Free Reactions

Although many mechanistically distinct methods via two-component reactions are described above, they require the use of expensive noble metals, often with expensive ligands, and external oxidants when needed to re-initiate the catalytic cycle. Meanwhile, the transition-metal-free approaches to functionalized 2-quinolones via two-component reaction have been widely studied. Some representative examples are presented in this section.

**Scheme 23:**
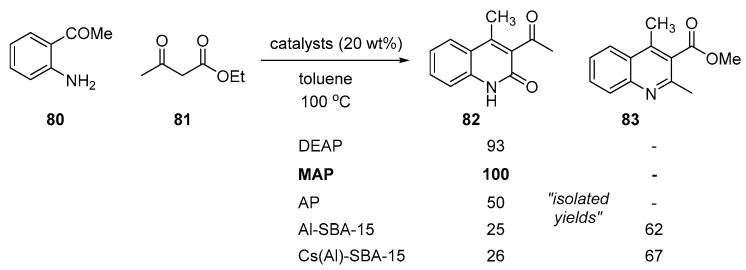
Friedländer reaction by basic mesoporous catalysts.

A classical Friedländer-type transformation could be accelerated by basic mesoporous catalysts. In 2009, Cejka addressed the use of basic heterogeneous catalysts supporting diethylaminopropyl (DEAP), methylaminopropyl (MAP), and aminopropyl (AP) for the synthesis of 3,4-disubstituted 2-quinolones [107]. The primary amine-supported catalyst (AP) resulted in lower yields than the secondary (MAP) or tertiary amine-supported (DEAP) catalyst. The MAP-based catalyst (20 wt %) showed the highest catalytic activity and complete selectivity toward 2-quinolone **82**, as depicted in Scheme 23. However, in this reaction, use of the acidic heterogeneous catalysts including Al-SBA-15 and its cesium salt Cs(Al)-SBA-15 was not efficient, resulting in reversed chemoselectivity.

**Scheme 24:**
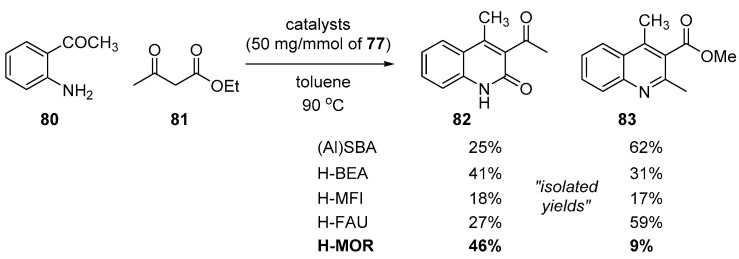
Friedländer reaction by bifunctional zeolites as Lewis and Brønsted acid catalyst.

In 2010, López-Peinado’s group reported a zeolite-promoted Friedländer reaction [108]. They developed a protocol using bifunctional zeolites with Lewis and Brønsted acid sites such as H-BEA, H-MFI, H-FAU, and H-MOR. The structure and morphology of the catalysts were characterized with IR spectra, X-ray diffraction (XRD), and scanning electron microscopy (SEM). The catalytic performance was evaluated in the condensation of 2-aminoaryl ketones **80** and ethyl acetoacetate **81** at elevated temperature; the results are summarized in Scheme 24. While the conversion and selectivity were not practically high, it is notable that H-MOR with higher Brønsted acid site concentration gave better selectivity towards 2-quinolone **82**. A similar approach using Amberlite Na sr1L as a strongly acidic catalyst was reported by Khan and coworkers [109].

**Scheme 25:**
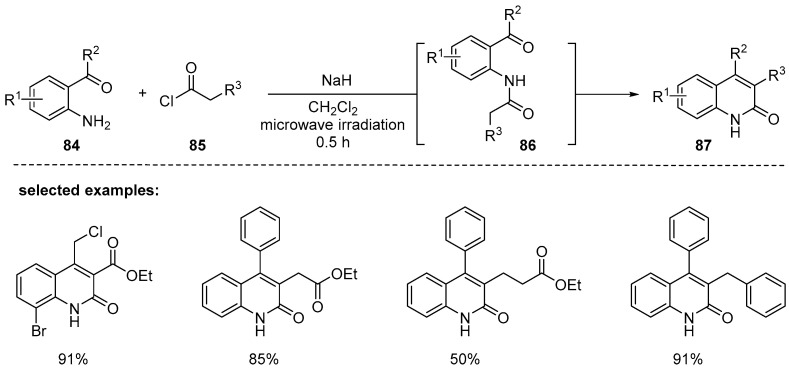
Synthesis of 3,4-disubstituted 2-quinolones by one-pot condensation promoted by microwave irradiation.

In 2014, Bui reported that the condensation reaction between substituted 2-aminobenzophenones **84** and acid chlorides **85** furnished 2-quinolinones **86** in the presence of a base under microwave irradiation [110]. They found that microwave irradiation is important in terms of reaction efficiency, shorter reaction time, and better conversion. The condensation reactions of simple benzophenones with ethyl malonyl chlorides proceeded smoothly to give **84** in good yields, although not relying on microwave irradiation. However, the reaction either of ketones possessing non-methyl alkyl groups at R^2^ or aliphatic acid chlorides required microwave irradiation for successful conversion. A strong base, such as sodium hydride (or sodium bis(trimethylsilyl)amide), was required to ensure annulation for the products **87** bearing alkyl substituents at the 3-position (Scheme 25).

**Scheme 26:**
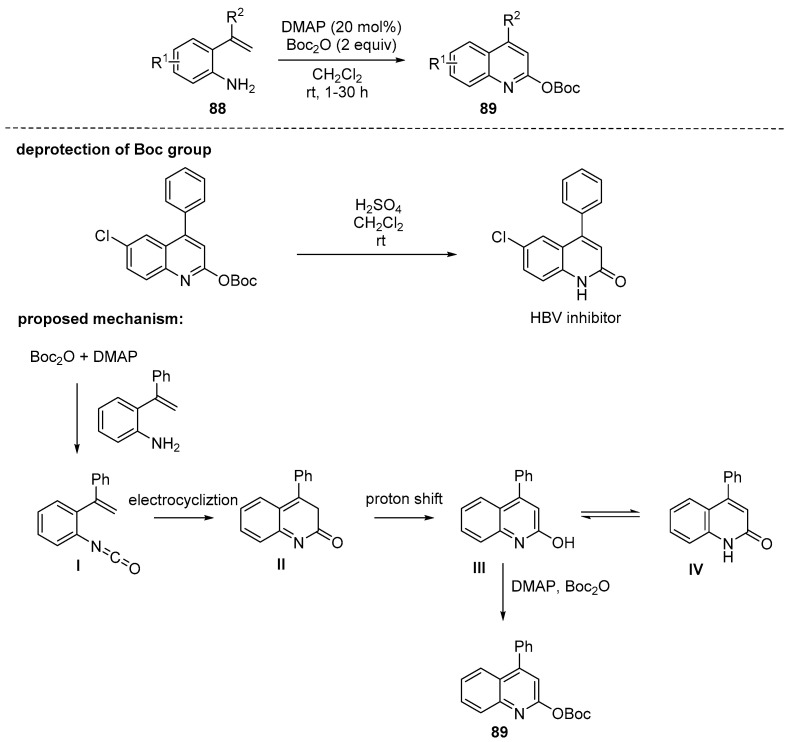
DMAP-catalyzed cyclization of Boc-anhydride of 2-alkenylanilines.

Deng and coworkers developed cascade annulation of 2-alkenylanilines in the presence of di-*t*-butyl carbonate (Boc_2_O) and a catalytic amount of 4-dimethylaminopyridine (DMAP) [111]. DMAP and Boc_2_O were used for transforming amines to the corresponding isocyanates **I** [112], which was converted to 2-hydroxyquinoline **III** or its tautomer **IV** by 6π-electrocyclization process. The electron-withdrawing and -donating functional groups in arenes were compatible to provide a series of *tert*-butyl quinolin-2-yl carbonates **89** in moderate to good yields. Deprotection of the *tert*-butyloxycarbonyl (Boc) group under H_2_SO_4_ condition could lead to 2-quinolones. A gram-scale reaction (1.56 g of starting material, R^1^ = H, R^2^ = Ph) was conducted and the desired product was successfully obtained in 92%. Moreover, one of the hepatitis B virus (HBV) inhibitors could be prepared in a 98% yield by only two-step simple transformation (Scheme 26).

**Scheme 27:**
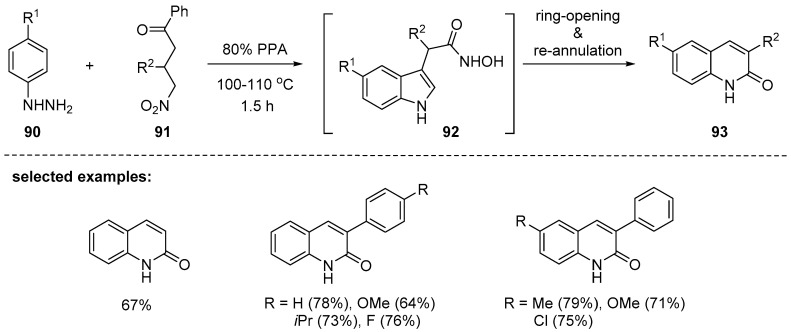
Formation of 2-quinolones in reaction between 4-nitroketones and hydrazines.

In 2014, Aksenov and coworkers reported an interesting synthesis of 3-substituted 2-quinolines based on the addition of the nucleophile, ring opening, and ring closure (ANRORC)-type ring expansion [113] of the hydroxamic acid **92**. The intermediate **92** was derived from the Fischer reaction of hydrazines **90** and 4-nitroketones **91** [114]. The optimized cyclization conditions used polyphosphoric acid (80% PPA) at 100–110 °C. The desired products were obtained with both electron-rich and -poor substituents at the C3 position of **88** including aryl- and alkyl groups and even with C3-nonsubstituted 4-nitroketones. On the basis of the previous report [113], a possible mechanism involving 5 to 6 ANRORC-type ring expansions of in situ generated hydroxamic acid intermediate **92** was proposed (Scheme 27).

**Scheme 28:**
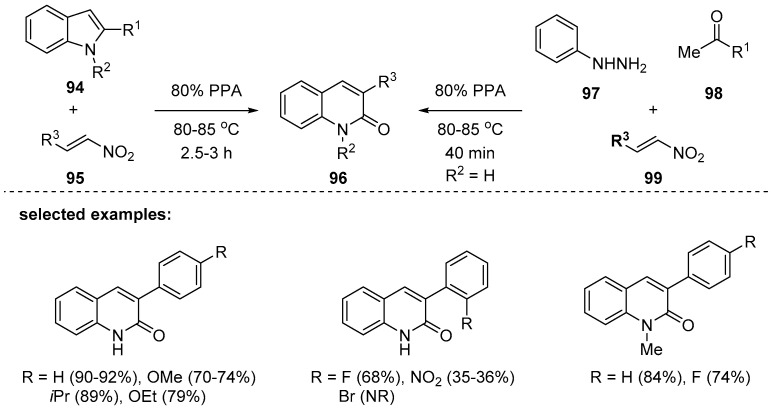
Synthesis of quinolones by reaction of 2-substituted indoles with 2-nitroalkenes under PPA.

In 2015, the same group proposed a simple synthesis of 3-substituted 2-quinolones **96** [115]. Hydroxamic acid intermediates similar to intermediate **92** in Scheme 27 were formed by electrophilic alkylation of indole **94** under polyphosphoric acid (PPA). Then, the hydroxamic acids were converted to 2-quinolinones via ring-opening and ring-closing cascade reactions. This reaction was sensitive to the steric environment of the nitrostyrenes **95**; for example, *ortho*-substituted nitrostyrenes resulted in low yields. Apart from the use of indoles **94** as substrates, the reaction was accomplished by three-component reaction using **97**, **98**, and **99**, in which in situ preparation of **94** was successful by Fischer indole synthesis (Scheme 28).

**Scheme 29:**
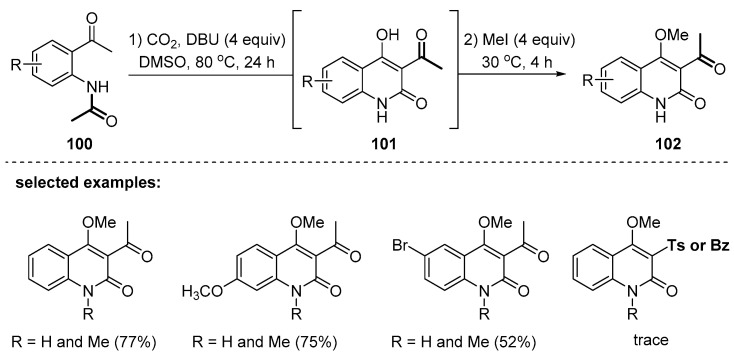
DBU-promoted annulation using *o*-acetamidoacetophenones and carbon dioxide.

In 2015, Zhang and coworkers disclosed a one-pot method for the preparation of 3-acetyl-4-methoxy-2-quinolones (Scheme 29) [116]. The two components used in the cascade reaction were *o*-acetamidoacetophenone **100** and carbon dioxide as a C2 carbonyl source. In the presence of strongly basic DBU (1,8-diazabicyclo[5.4.0]undec-7-ene), *N*-acetyl substrates **100** were smoothly transformed to the corresponding quinolones **101** with the C3-acetyl group. The acetyl functionality was proposed to involve migration from nitrogen to the C3 position in the presence of DBU. However, *N*-benzoyl- or tosyl-protected *o*-aminoacetophenones were not compatible with the reaction conditions. The 4-hydroxy group of **101** was in situ methylated in the same flask to afford **102**.

**Scheme 30:**
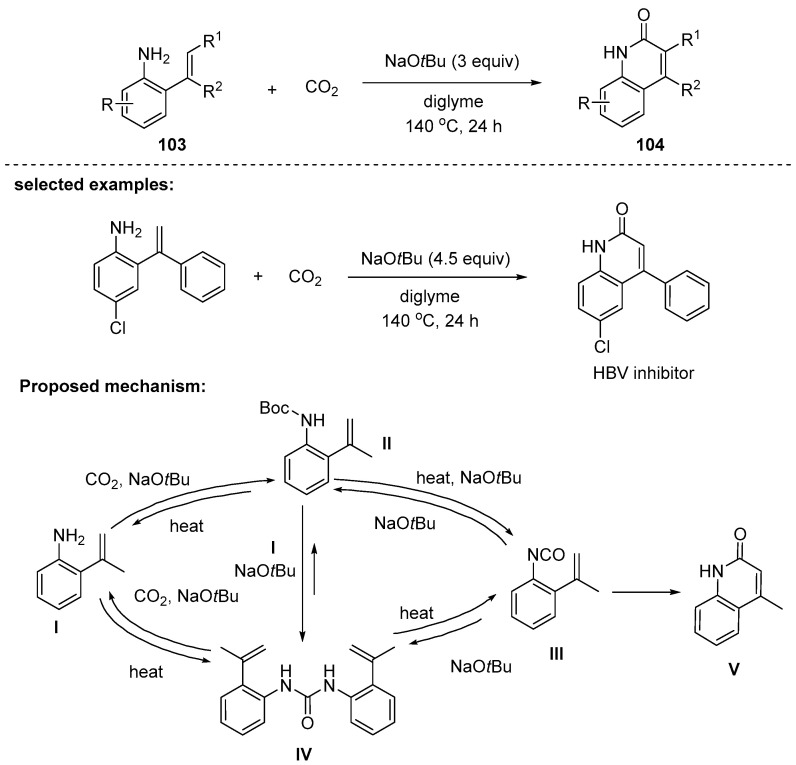
Lactamization of 2-alkenylanilines toward substituted 3,4-substituted 2-quinolones.

In 2016, Yu and coworkers published a practical method of base-mediated lactamization of sp^2^ C–H bond with CO_2_ to access 2-quinolones **104** in good to excellent yields [117]. Note that carbon dioxide was used as a carbonyl source of quinolone scaffolds. The reaction with diglyme as a solvent was most effective and the use of alkali metal *tert*-butoxides as a base gave better yields than those with other bases (NaH, KOH, and DBU). Several alkali metal *tert*-butoxides were screened and NaO*t*Bu was found to be superior to other bases, such as LiO*t*Bu and KO*t*Bu, providing a 95% yield at 140 °C. Various substituted anilines with *ortho*-alkenyl moiety were successfully converted to 4-substituted 2-quinolones in moderate to excellent yields. In particular, the *ortho* α-styrenyl anilines reacted with CO_2_ to afford 4-aryl-2-quinolones. The synthetic utility of the method was demonstrated by the synthesis of 6-chloro-4-phenyl-2-quinolone (HBV inhibitor). The proposed mechanistic pathway is depicted in Scheme 30. During the mechanistic studies, through some control experiments, the compounds such as **II**–**IV** were observed. Although not conclusive, the key intermediate to allow for cyclization seems to be the isocyanate **III**.

## 3. Three-Component Reactions

### 3.1. Pd-Catalyzed Reactions

**Scheme 31:**
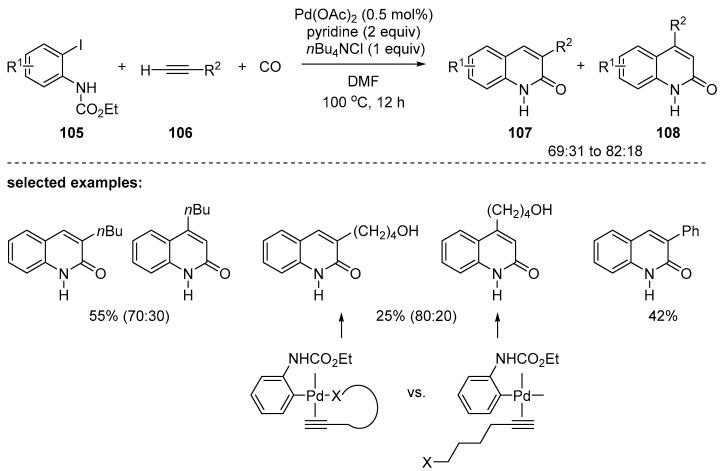
Palladium-catalyzed annulation of terminal alkynes.

Palladium-catalyzed carbonylative annulation of alkynes is a representative synthetic pathway to prepare 2-quinolones. In 2003, the Larock research group reported the first palladium-catalyzed synthesis of 2-quinolones using three components, an *o*-iodoaniline **105**, a terminal alkyne **106**, and carbon monoxide gas [118]. Mixtures of 3- and 4-substituted 2-quinolones, **107** and **108**, were obtained when the terminal alkyne bore more than four carbon alkyl chains. Moreover, 3-substituted 2-quinolones were predominant over 4-substituted ones and the ratios of the two regioisomers were greater than 69:31 in all cases (Scheme 31). Interestingly, the terminal alkynes with alkyl alcohol, ester, or nitrile provided the desired products in better regioselectivity up to 81:19. These results can be explained by the fact that the palladium complex is further stabilized by additional coordination of the palladium atom with the functional group of the alkyne. The reaction with phenyl acetylene provided the product in excellent regioselectivity, even though polymeric byproducts were formed significantly.

**Scheme 32:**
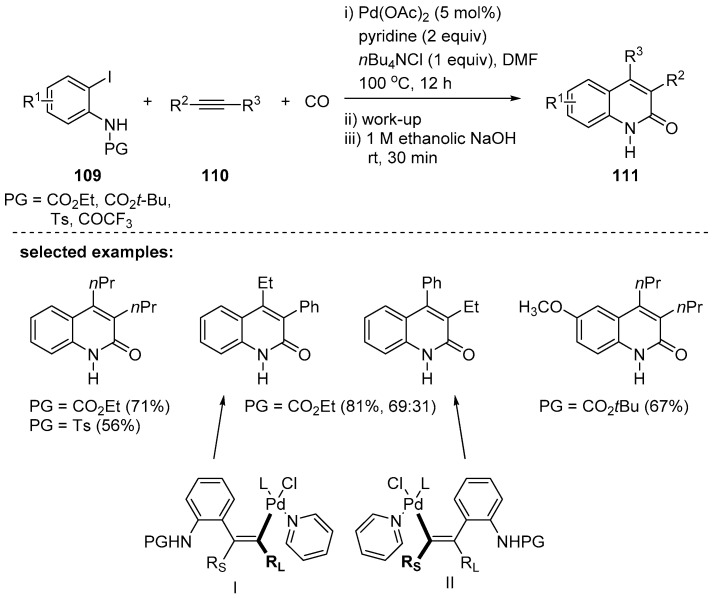
Palladium-catalyzed annulation of internal alkynes.

Furthermore, 3,4-disubstituted 2-quinolones **111** could be prepared using internal alkynes **110** in the presence of carbon monoxide gas through palladium-catalyzed carbonylative annulation [119]. *N*-Protected *o-*iodoanilines **109** are utilized in this process, and the choice of protecting groups was important. After completion of the reaction, the protecting groups were removed by treatment of 1 M ethanolic NaOH to provide the unprotected 2-quinolones. Symmetric internal alkynes with alkyl, (hetero)aryl, alkoxyl, and hydroxyl groups gave the desired products in moderate to good yields. However, mixtures of regioisomers with poor selectivity due to two possible modes of alkyne insertion were obtained when unsymmetrical alkynes were employed (Scheme 32). It is suggested that the regioselectivity is determined based on the steric factors of two different substituents of alkynes. Less steric repulsion is expected in the palladium complex **I** than **II** between the larger substituent of the alkyne and palladium because of the longer palladium–carbon bond compared to the carbon–carbon bond in these two complexes. Carbonylative reactions using carbon monoxide gas have some drawbacks due to high toxicity and difficulties in the handling of gaseous carbon monoxide. As an alternative to carbon monoxide gas, a solid carbon monoxide source, Mo(CO)_6_, has been used in carbonylative reactions, and carbon monoxide is generated in situ during the course of the reactions.

**Scheme 33:**
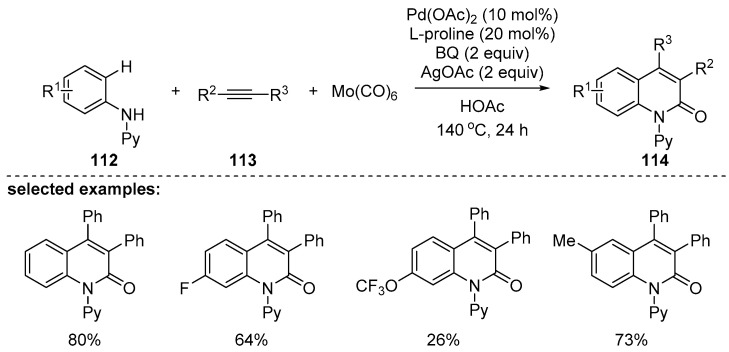
Carbonylative [3+2+1] annulation by C–H activation.

In 2004, the Wu group [120] reported the synthesis of 2-quinolones via palladium-catalyzed carbonylative annulation of anilines and internal alkynes with Mo(CO)_6_ as a solid carbon monoxide source. Mo(CO)_6_ could successfully replace the carbon monoxide gas and various *N*-pyridine protected 2-quinolones **114** were successfully synthesized by carbonylative [3+2+1] annulation of *N*-arylpyridine-2-amines **112** with internal alkynes **113** by a C–H activation (Scheme 33). Proper choice of oxidant (BQ) and co-oxidant (AgOAc) was important, and the desired products were obtained in good yields with the optimized conditions.

**Scheme 34:**
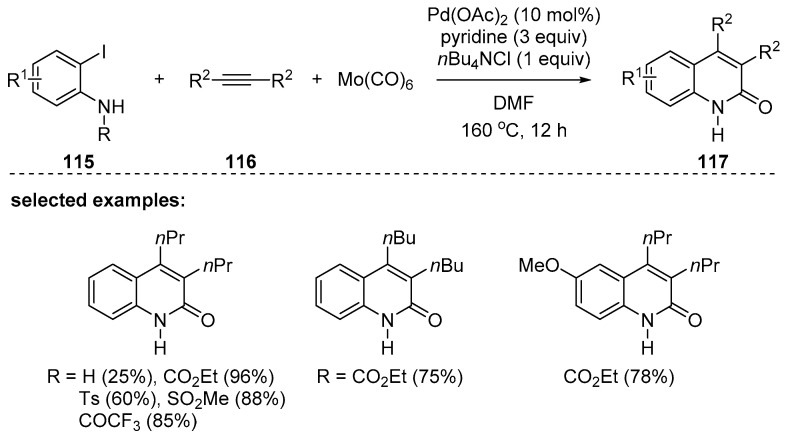
Annulation of *N*-substituted *o*-iodoanilines.

The Otaredi-Kashani group also reported CO gas-free synthesis of 2-quinolones [121]. The palladium-catalyzed carbonylative annulation with *N*-protected *o*-iodoanilines **115** and internal alkynes **116** in the presence of molybdenum hexacarbonyl, Mo(CO)_6_, provided 3,4-disubstituted 2-quinolones **117** in good yields (Scheme 34). Due to the low yields with unprotected anilines, various *N*-protecting groups such as -CO_2_Et, -Ts, -Ms, and -COCF_3_ of the aniline were explored, and deprotection proceeded in the course of the reaction to provide the unprotected 2-quinolones **117**. Unfortunately, only a trace amount of the desired product was obtained when using diphenylacetylene because of homocoupling and polymerization.

**Scheme 35:**
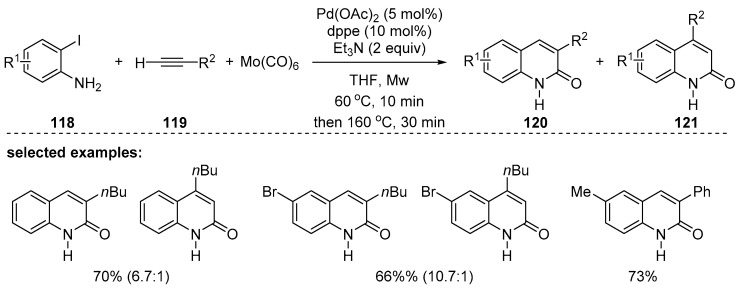
Microwave-assisted carbonylative cyclization.

In 2010, Xiao and coworkers reported microwave-assisted palladium-catalyzed carbonylative cyclization reactions using *o*-iodoanilines **118**, terminal alkynes **119**, and a solid carbon monoxide source, [Mo(CO)_6_] [122]. Compared to thermal reaction conditions, this reaction proceeded rapidly and 3- or 4-substituted 2-quinolones were efficiently synthesized. However, mixtures of 3- and 4-substituted 2-quinolones, **120** and **121**, were obtained in most cases (Scheme 35).

**Scheme 36:**
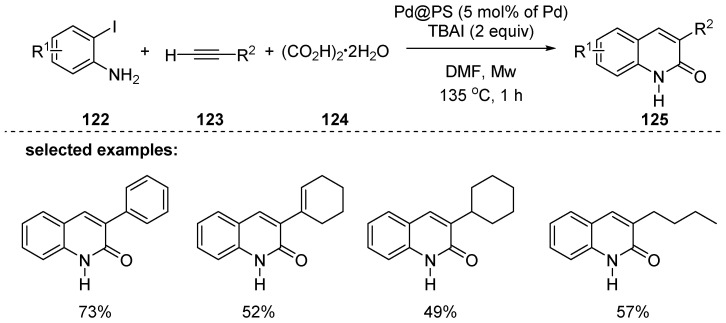
Regioselective synthesis of 3-substituted 2-quinolone using Pd@PS.

As an alternative to carbon monoxide gas, oxalic acid dihydrate **124**, (COOH)_2_·2H_2_O, was used as an in situ generated carbon monoxide source for the synthesis of 2-quinolones **125** (Scheme 36). Das and coworkers used heterogeneous polystyrene-supported palladium, Pd@PS, as a catalyst to effectively hold the (COOH)_2_·2H_2_O under microwave-assisted reaction conditions [123]. Interestingly, the Pd@PS catalyst can be recycled up to four times without loss of activity. Moreover, 3-substituted 2-quinolones **125** were obtained in a regioselective manner and 4-substituted regioisomers were not detected.

**Scheme 37:**
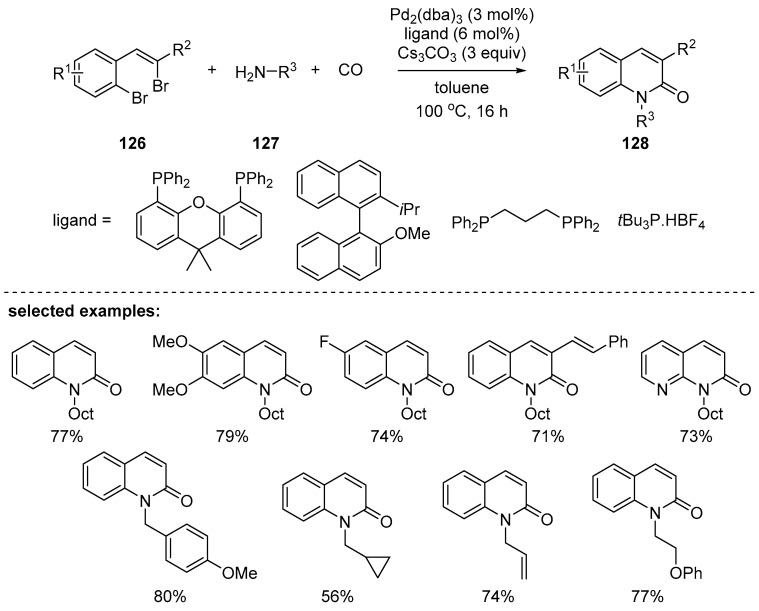
Cascade alkenyl aminocarbonylation and intramolecular aryl amidation.

A cascade reaction of palladium-catalyzed intermolecular aminocarbonylation followed by intramolecular amidation was reported by Willis’ group in 2009 [124]. Here, 2-(2-bromoalkenyl)aryl bromide **126** effectively reacted with a variety of primary amines **127** in the presence of atmospheric pressure of carbon monoxide under the palladium-catalyzed reaction conditions. Depending on the substituents on the amines, various *N*-alkyl-, aryl-, or carbonylated 2-quinolones **128** were obtained in moderate to good yields (Scheme 37). Between two possible bromides, an alkenyl- and aryl bromide, of 2-(2-bromoalkenyl)aryl bromide **126**, the alkenyl bromide reacts first with the palladium catalyst followed by carbonylation and then amination to give the corresponding 2-quinolones.

**Scheme 38:**
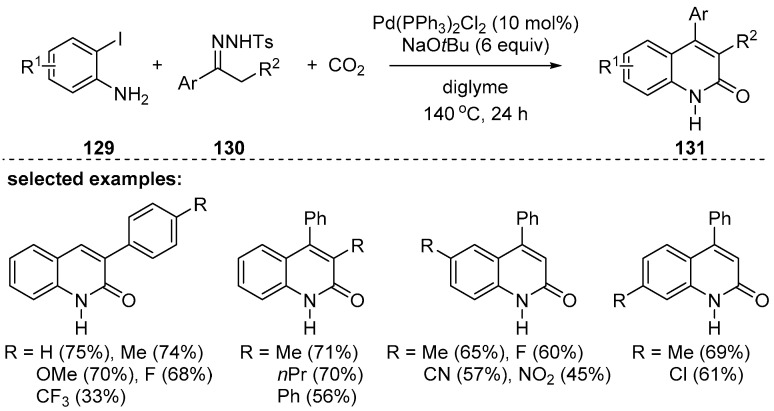
Synthesis of 4-aryl-2-quinolinones in the presence of CO_2_ gas.

Carbon dioxide, a greener source of a carbonyl moiety compared to hazardous CO gas, can be also used as a carbonyl fragment in the synthesis of 2-quinolones. Cheng and coworkers reported the synthesis of 4-aryl 2-quinolones **131** using *o*-iodoanilines **129**, *N*-tosylhydrazones **130**, and carbon dioxide in the presence of a palladium catalyst [125]. In the presence of a base, *N*-tosylhydrazones converts to diazo substrates to generate palladium carbene intermediate, whose catalytic cycle was followed by migratory insertion, reaction with CO_2_, and 6π-electrocyclic reaction to provide the desired 2-quinolone products. A variety of 4-aryl substituted or 3,4-disubstituted 2-quinolones were obtained based on the substitution patterns of *N*-tosylhydrazones in moderate to good yields (Scheme 38).

### 3.2. Other Transition-Metal-Catalyzed Three-Component Reactions

**Scheme 39:**
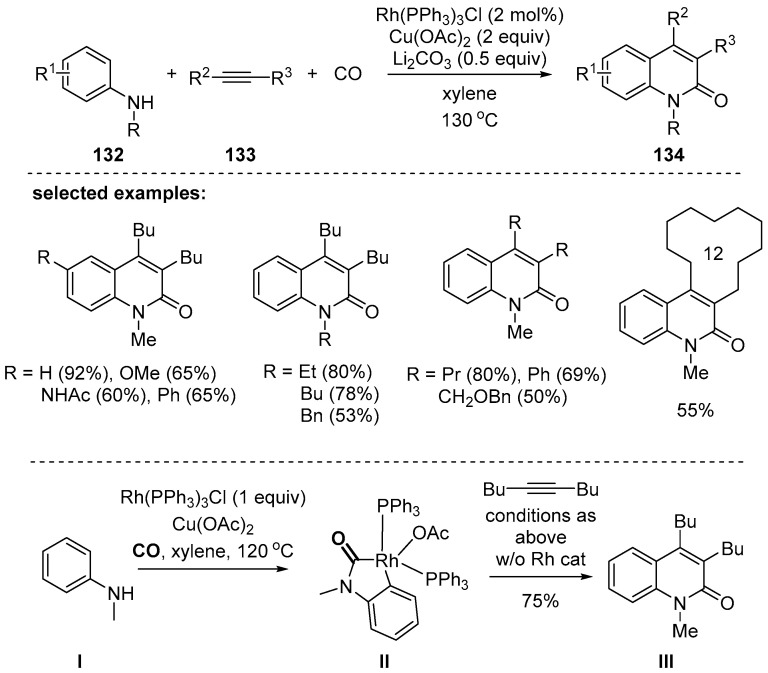
C–H activation of anilines with alkynes and CO gas.

In 2015, Jiao and coworkers reported Rh-catalyzed 2-quinolone synthesis with anilines **132**, internal alkynes **133**, and carbon monoxide through C–H activation [126]. Cu(OAc)_2_ was used as an efficient oxidant in this process and Li_2_CO_3_ was used as a base. With the use of symmetric internal alkynes, 3,4-disubstituted 2-quinolones **134** were obtained in moderated to high yields. A broad range of functional groups were tolerated. By control experiment, rhodacycle **II** was prepared from the compound **I** in the presence of a stoichiometric amount of Rh(PPh_3_)_3_ and the structure **II** was determined by X-ray diffraction. Reaction between the rhodacycle **II** and dibutylacetylene provided the corresponding 2-quinolone **III** (Scheme 39).

**Scheme 40:**
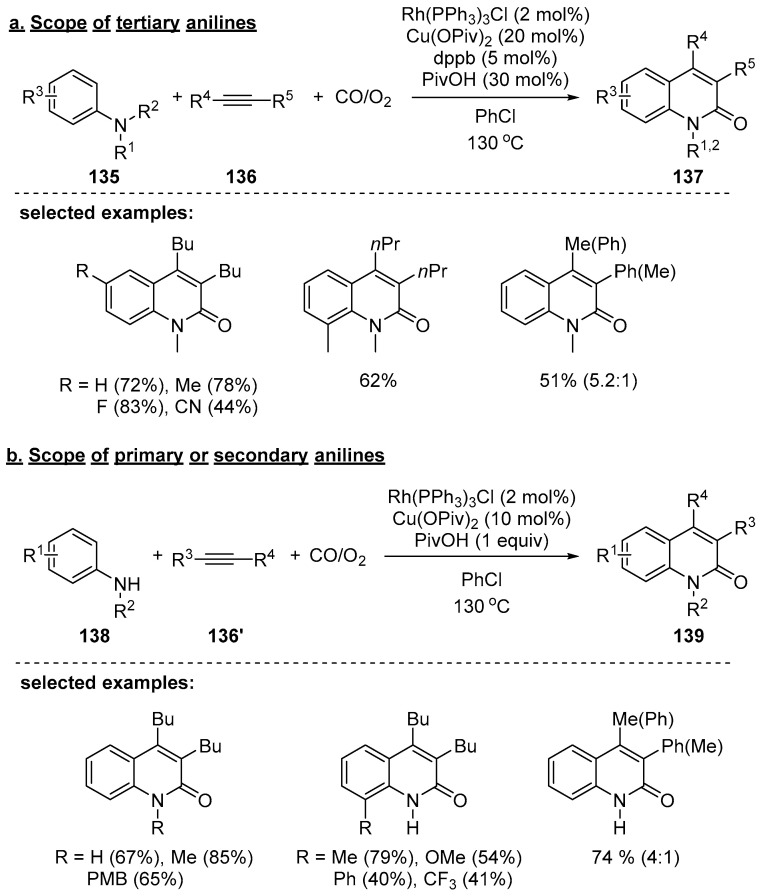
Rh-catalyzed aerobic oxidative cyclization of simple anilines with internal alkynes.

Jiao and coworkers also reported Rh-catalyzed aerobic oxidative synthesis of 2-quinolones with anilines **135**, internal alkynes **136**, and carbon monoxide [127]. Even though synthetic challenges exist due to the sensitivity of amines to strong oxidants, primary- and tertiary amines were successfully utilized in this Rh-catalyzed aerobic oxidative cyclization. In the proposed catalytic cycle, Rh(I) can be oxidized to Rh(III) by Cu(II); then, the resulting Cu(I) catalyst is re-oxidized to Cu(II) in the presence of O_2_ and carbon monoxide. By the DFT calculation, a rhodium catalyst is required for the efficient transformation, and palladium or iridium were found to be inefficient in this process because of a high energetic barrier. During optimization of the catalytic system, the best ratio of CO/O_2_ was 2:1 (1 atm) for tertiary amines and 4:1 (1 atm) for primary/secondary amines. It is notable that a catalytic amount of dppb was required in cases using tertiary anilines. When asymmetric aryl alkyl alkynes were used, mixtures of regioisomers were obtained in good yields with moderate regioselectivity (Scheme 40a). When primary and secondary anilines were used, the corresponding 2-quinolones were generated in moderated to good yields as well (Scheme 40b).

**Scheme 41:**
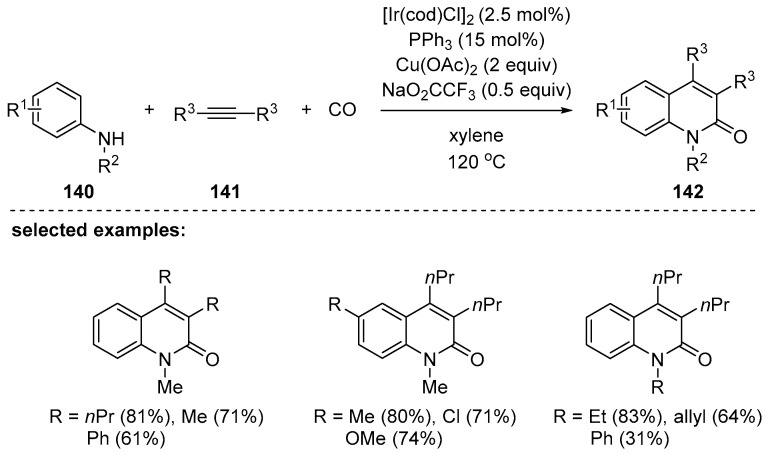
Synthesis of 2-quinolinone by Ir-catalyzed three-component reactions.

Ir-Catalyzed C–H/N–H activation with anilines **140**, internal alkynes **141**, and carbon monoxide for the synthesis of 2-quinolones was reported by Wu’s group in 2016 [128]. Even though the reaction required high pressure of carbon monoxide (30 bar), a wide range of alkyl-, aryl-, or heteroaryl 3,4-disubstituted 2-quinolone derivatives were obtained by Ir-catalyzed carbonylative annulation (Scheme 41). The developed reaction featured no requirements for preactivation or directing groups. The proposed mechanism included a five-membered iridacycle as the key intermediate, which is similar to the rhodacycle reported by Jiao et al. [127].

**Scheme 42:**
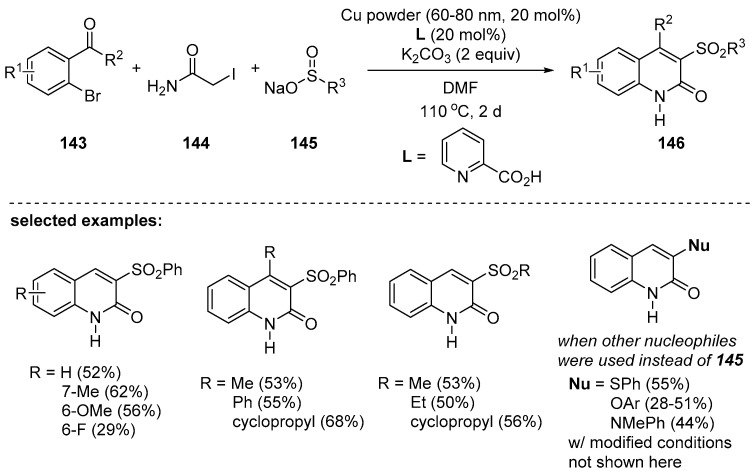
Cu-powder-catalyzed one-pot synthesis of 3- and/or 4-substituted 2-quinolones.

Recently, Kim and Lim reported Cu-catalyzed synthesis of 2-quinolones by S_N_2, Knoevenagel condensation, and C–N bond forming process [129]. Functionalization with various sulfonyl groups on the 3-position of 2-quinolones was enabled by employing various aryl- or alkylsulfinate sodium salts (Scheme 42). In addition to sulfur(II), use of other nucleophiles such as a phenoxide, thiolate, and an aniline was also demonstrated. Compared to other three-component reactions, the carbonyl moiety of 2-quinolones stems from amides **141**, not from carbon monoxide or its equivalents. Even though longer reaction time (2 days) was required, 3,4-disubstituted 2-quinolones could be synthesized in a straightforward manner.

## 4. Miscellaneous

### 4.1. Four-Component Reactions and Related Protocols

Whereas numerous examples using two- or three-component reactions have been reported for 2-quinolone synthesis, more complicated systems using four, five, or six components are rare. The established protocol for the multi-component reaction represents a Ugi four-component reaction that uses an amine, isocyanide, aldehyde, and carboxylic acid. This cascade reaction can afford 1,4-bisamide products. Several interesting procedures employing the Ugi reaction were identified, as described below, with more than three components to construct 2-quinolones. Some examples covered in this section are not directly related to the one-pot reaction; however, they are potentially important as one-pot protocols which can give distinct *N*-substitution of 2-quinolones.

**Scheme 43:**
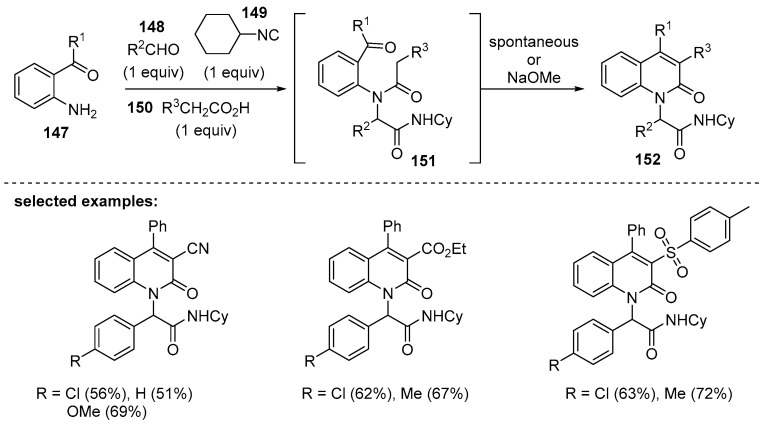
Synthesis of multisubstituted 2-quinolones via four-component reaction.

In 2004, Torroba and coworkers reported a novel synthetic method for 3,4-disubstituted 2-quinolones that features a four-component reaction [130]. This one-pot cascade reaction was enabled by tandem Ugi/Knoevenagel reactions using 2-aminophenylketones **147**, aldehydes **148**, cyclohexylisocyanide **149**, and malonic or sulfonylacetic acid derivatives **150** (Scheme 43). The first step involves the Ugi four-component reaction to form the bisamides **151** [131,132]. Then, **151** was cyclized by Knoevenagel condensation to afford the densely functionalized 2-quinolone derivatives **152**. It was notable that spontaneously annulated products were observed when R^3^ was nitrile or ester, whereas the bisamides themselves were isolated when R^3^ was arylsulfonyl groups.

**Scheme 44:**
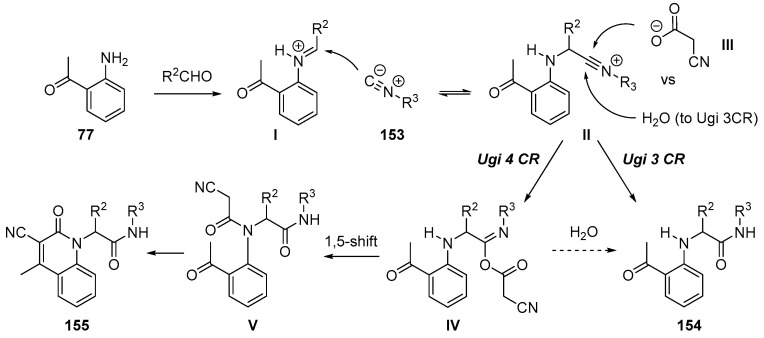
McCluskey’s studies on four-component reaction.

The related studies were found in an article in 2011 [133]. McCluskey and coworkers reported the formation of bisamides **154** by unexpected three-component Ugi reaction during the library synthesis of 2-quinolone derivatives. It was notable that the nucleophilicity of the carboxylic acid affected the selectivity towards three- vs. four-component reactions. As demonstrated in their studies, cyanoacetic acid gave a mixture of both Ugi three- and four-component reaction products. Interestingly, the four-component Ugi products were spontaneously converted to the corresponding quinolones **155** while the three-component Ugi products were isolated as monoamides **154**. A reaction mechanism was proposed; a route to three-component reaction product **154** was suggested to be the addition of H_2_O to **II**, but hydrolysis of **IV** cannot be excluded (Scheme 44).

**Scheme 45:**
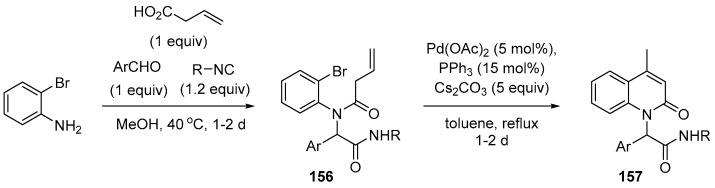
Preparation of 2-quinolones using four-component adduct via Heck reaction.

The next variant of this chemistry was reported by Foroumadi and coworkers [134]. Intramolecular Heck reaction was utilized with the precursors **156** prepared by Ugi four-component reaction. By employing 4-butenoic acid in the Ugi reaction, the requisite alkene moiety for the Heck reaction was installed and it reacted with aryl halide, affording *N*-alkyl 4-methyl-2-quinolone derivatives **157** after double bond isomerization (Scheme 45). The optimized conditions of the Heck reaction involved Pd(OAc)_2_, PPh_3_, and Cs_2_CO_3_ in refluxing toluene.

**Scheme 46:**
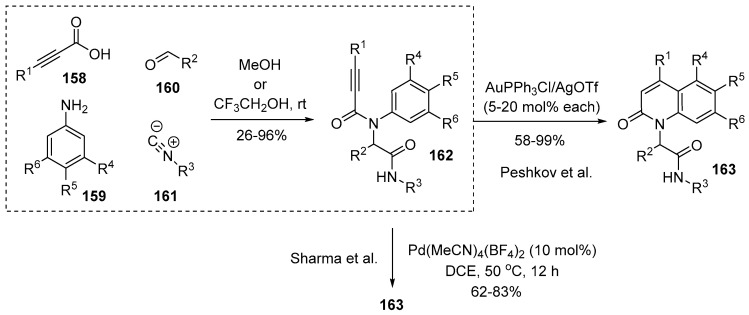
π-Acid-catalyzed cyclization of four-component adducts containing alkynes.

A similar four-component reaction was utilized by Peshkov and coworkers. Alkynic acids were used to form key intermediates **162** for preparation of 4-substituted 2-quinolones **163** [135]. While several types of hydroarylation reaction by alkyne activation to construct the lactam nucleus of 2-quinolone were reported, the authors sought diversity of *N*-substituents in this transformation; their choice for *N*-variation was the Ugi reaction. As illustrated in Scheme 46, the diversely *N*-substituted precursors **162** were prepared for the Au-catalyzed annulation. Recent examples of utilizing the Ugi adducts were reported by Sharma and coworkers in 2019 [136]. In their work, a similar 6-endo-dig hydroarylation was designed by employing cationic Pd-catalysts to furnish 2-quinolones.

**Scheme 47:**
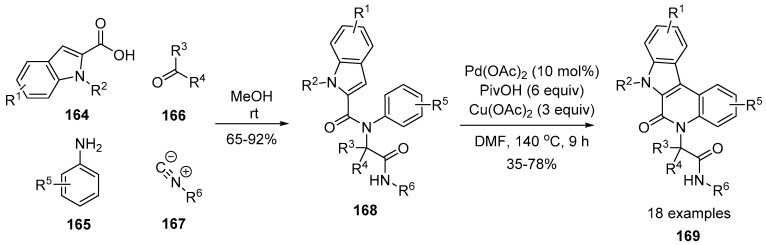
Preparation of indoloquinolones using four-component reaction and C–H activation.

The most recent example of the Ugi reaction was presented by Dömling and coworkers [137]. Indole-2-carboxylic acids **164** can be used as a platform for the preparation of the tetracyclic scaffolds **166**. The key intermediates **168** were subjected to Pd-catalyzed dual C–H bond functionalization reactions and, accordingly, fused tetracyclic indoloquinolones **169** were obtained in moderate yields (Scheme 47). The use of the readily available building blocks as starting materials is an important feature of this method, which likely allows for a modular approach. Moreover, the prepared material **169** was evaluated in an in silico docking model to find potential kinase inhibitors.

### 4.2. Recent Approaches to Fused 2-Quinolones

**Scheme 48:**
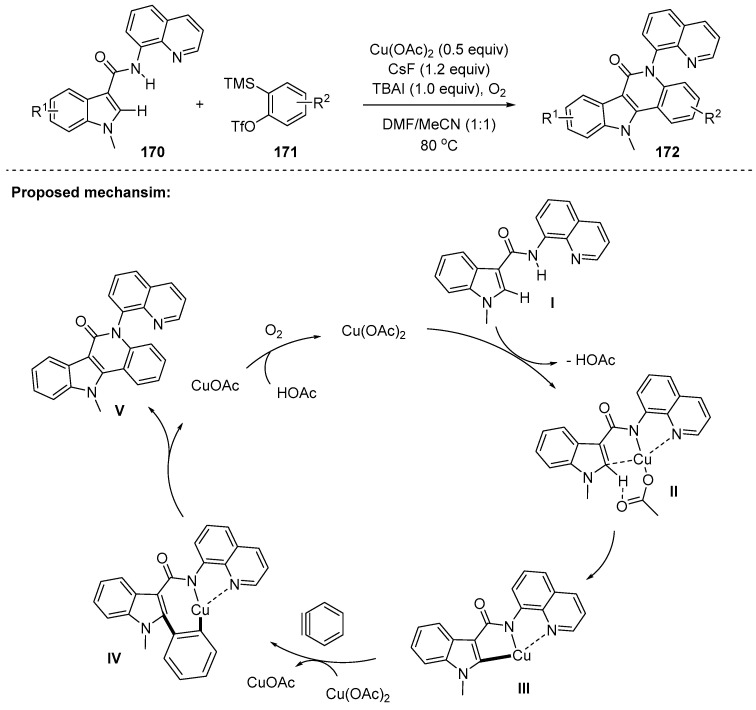
Synthesis of indoloquinolones via double N–H/C–H activation.

As demonstrated in two-component reactions, arynes could participate in the cyclization via a cascade C–H/N–H bond activation. In 2018, Zhang and coworkers developed the protocol to furnish tetracyclic indoloquinolones **172** via Cu(II)-mediated annulation using *N*-quinolyl indole-3-carboxamides **170** and in situ generated arynes from benzyne precursors **171** [138]. In this way, medicinally important indoloquinoline derivatives were readily accessible; as an application of the method, formal synthesis of the indoloquinoline alkaloid such as isocryptolepine (natural antimalarial) was accomplished. The proposed mechanism is depicted in Scheme 48. Through some control experiments including a primary intermolecular KIE (value = 1.3) and radical trapping with TEMPO, a SET mechanism was excluded. Instead, the key concerted metalation/deprotonation (CMD) pathway was proposed to form the corresponding metallacycle **III**, which in turn underwent carbocupration with the aryne (**III→IV**) and reductive elimination (**IV→V**).

**Scheme 49:**
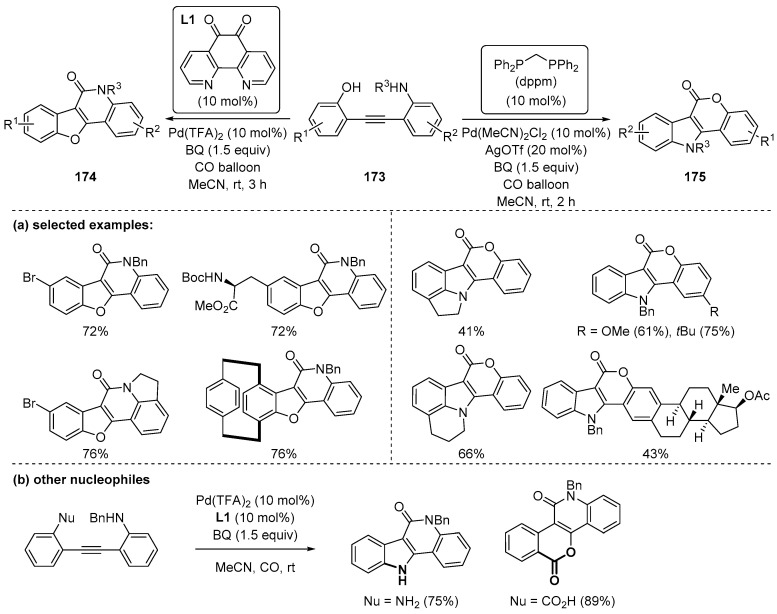
Ligand-controlled regioselective synthesis of 3,4-fused-2-quinolones.

In 2018, Jiang and coworkers developed the regiodivergent carbonylation of dinucleophilic diarylalkynes **173** containing an alcohol and an amine; regioselective control was enabled by a Pd(II) catalyst and bidentate ligands [139]. Depending on the ligands, different regioisomers were synthesized; benzofuro[3,2-c]quinolones **174** were obtained by the use of an electron-deficient 1,10-phenanthroline-5,6-dione (**L1**) and indolo[3,2-c]coumarins **175** were obtained by use of a sterically bulky and electron-rich ligand, dppm. Due to the merit of divergent method, many structurally fascinating compounds were exemplified (Scheme 49a). It is notable that this method was also applicable to the substrates containing an amine and carboxylic acid as alternative nucleophiles instead of an alcohol. The reactions using them afforded indoloquinolones, pyranoquinolones, and 3,4-fused pentacyclic 2-quinolones in one pot (Scheme 49b).

## 5. Conclusions and Outlook

In this review, we give an overview of recent synthetic efforts for the construction of 2-quinolone scaffolds. The contents were classified by the number of reaction components and the type of transition metals when applied. The early synthetic chemistry of 2-quinolones consists basically of variations on two types of reactions: Friedländer reaction and Knorr reaction. However, recent advances in new bond-forming reactions led to new approaches via unconventional designs. It should be noted that non-classical access to 2-quinolones was greatly expanded after the introduction of the Pd-catalyzed two-component reaction by R.F. Heck in 1978. In this process, *ortho*-iodoanilines and 1,2-disubstituted olefins were elaborated into 2-quinolones through the palladium-catalyzed vinylic substitution reaction.

In many cases, for the synthesis of 2-quinolones, the palladium-catalyzed cross-coupling reactions between two components appeared to be crucial methods. The palladium-catalyzed cross-coupling chemistry has been further combined with the recently evolved palladium-catalyzed C–H activation and tandem coupling process for the synthesis of more complex and multi-substituted 2-quinolones. While palladium is still the most exploited transition metal for the synthesis of 2-quinolones, many efforts have been directed toward finding other transition metals. Significant advances have been made in the copper-catalyzed two-component reactions over the last few decades by replacing Pd-catalyzed reactions; Ullmann type C–N bond forming reactions were adopted for synthesis of the lactam nucleus in 2-quinolones. Besides these, other transition metals such as Rh, Ru, Ag, and Ir were also successfully used as catalysts of two-component reactions, although only a few examples were demonstrated.

In 2003, Larock and coworkers reported a seminal work for the synthesis of 2-quinolones via Pd-catalyzed three-component reaction using *o*-aniline, alkyne, and carbon monoxide (CO) gas. Since then, the preparation of 2-quinolones by three-component reaction using other carbonyl equivalents was investigated. Among various carbonyl precursors including Mo(CO)_6_, oxalic acid hydrate, and carbon dioxide, molybdenum hexacarbonyl [Mo(CO)_6_] was the most useful source when reaction scale-up required a solid source of carbon monoxide. In these reaction settings, various transition metals (Pd, Cu, Rh, etc.) were employed with the complementary reaction scope to prepare multi-substituted 2-quinolone derivatives.

Although only one approach by the Ugi four-component reaction was found to provide 2-quinolones, it is believed that much more opportunities remain to develop novel methods using other types of four- or five-component reactions.

For decades, a series of pharmacologically important derivatives of 2-quinolones have been synthesized, mostly through facile two-component and three-component reactions. Not surprisingly, the development of synthetic methodologies of 2-quinolones is mostly gaining new momentum due to advances in transition metal chemistry. Nonetheless, the literature regarding 2-quinolone synthesis for medicinal purposes shows that only a few protocols are considered in the route-scouting from a practical point of view. Critical challenges still exist in the construction of a 2-quinolone core with readily available chemical feedstock and in the efficient introduction of substituents. We anticipate that the recent development of electrochemistry as well as photoredox chemistry will bring forth a range of improved transformations to functionalized 2-quinolones previously thought difficult to access.

## References

[1] Pham T.D.M., Ziora Z.M., Blaskovich M.A.T. (2019). Quinolone antibiotics. Med. Chem. Commun..

[2] Xing G., Pan L., Yi C., Li X., Ge X., Zhao Y., Liu Y., Li J., Woo A., Lin B. (2019). Design, synthesis and biological evaluation of 5-(2-amino-1-hydroxyethyl)-8-hydroxyquinolin-2(1*H*)-one derivatives as potent β_2_-adrenoceptor agonists. Bioorg. Med. Chem..

[3] Aparici M., Carcasona C., Ramos I., Montero J.L., Otal R., Ortiz J.L., Cortijo J., Puig C., Vilella D., De Alba J. (2019). Pharmacological profile of AZD8871 (LAS191351), a novel inhaled dual M_3_ receptor antagonist/β_2_-adrenoceptor agonist molecule with long-lasting effects and favorable safety profile. J. Pharmacol. Exp. Ther..

[4] Ramos I., Aparici M., Letosa M., Puig C., Gavaldà A., Huerta J.M., Espinosa S., Vilella D., Miralpeix M. (2018). Abediterol (LAS100977), an inhaled long-acting β_2_-adrenoceptor agonist, has a fast association rate and long residence time at receptor. Eur. J. Pharmacol..

[5] Ge X., Woo A.Y.-H., Xing G., Lu Y., Mo Y., Zhao Y., Lan Y., Li J., Yan H., Pan L. (2018). Synthesis and biological evaluation of β_2_-adrenoceptor agonists bearing the 2-amino-2-phenylethanol scaffold. Eur. J. Med. Chem..

[6] Aparici M., Carcasona C., Ramos I., Montero J.L., Ortiz J.L., Cortijo J., Puig C., Vilella D., Doe C., Gavaldà A. (2017). Pharmacological preclinical characterization of LAS190792, a novel inhaled bifunctional muscarinic receptor antagonist/β_2_-adrenoceptor agonist (MABA) molecule. Pulm. Pharmacol. Ther..

[7] Hughes A.D., Chen Y., Hegde S.S., Jasper J.R., Jaw-Tsai S., Lee T.-W., McNamara A., Pulido-Rios M.T., Steinfeld T., Mammen M. (2015). Discovery of (*R*)-1-(3-((2-chloro-4-(((2-hydroxy-2-(8-hydroxy-2-oxo-1,2-dihydroquinolin-5-yl)ethyl)amino)methyl)-5-methoxyphenyl)-amino)-3-oxopropyl)-piperidin-4-yl [1,1′-biphenyl]-2-ylcarbamate (TD-5959, GSK961081, Batefenterol): First-in-class dual pharmacology multivalent muscarinic antagonist and β_2_ agonist (MABA) for the treatment of chronic obstructive pulmonary disease (COPD). J. Med. Chem..

[8] Jones L.H., Burrows J., Feeder N., Glossop P., James K., Jones R.M., Kenyon A.S., Patel S., Roberts D.F., Selby M.D. (2015). Molecular hybridization yields triazole bronchodilators for the treatment of COPD. Bioorg. Med. Chem. Lett..

[9] Tang Q., Zhai X., Tu Y., Wang P., Wang L., Wu C., Wang W., Xie H., Gong P., Zheng P. (2016). Synthesis and antiproliferative activity of 6,7-disubstituted-4-phenoxyquinoline derivatives bearing the 2-oxo-4-chloro-1,2-dihydroquinoline-3-carboxamide moiety. Bioorg. Med. Chem. Lett..

[10] Han S.-Y., Choi J.W., Yang J., Chae C.H., Lee J., Jung H., Lee K., Ha J.D., Kim H.R., Cho S.Y. (2012). Design and synthesis of 3-(4,5,6,7-tetrahydro-3*H*-imidazo[4,5-c]pyridin-2-yl)-1*H*-quinolin-2-ones as VEGFR-2 kinase inhibitors. Bioorg. Med. Chem. Lett..

[11] Chen J., Peng H., He J., Huan X., Miao Z., Yang C. (2014). Synthesis of isoquinolinone-based tricycles as novel poly(ADP-ribose)polymerase-1 (PARP-1) inhibitors. Bioorg. Med. Chem. Lett..

[12] Hannigan K., Kulkarni S.S., Bdzhola V.G., Golub A.G., Yarmoluk S.M., Talele T.T. (2013). Identification of novel PARP-1 inhibitors by structure-based virtual screening. Bioorg. Med. Chem. Lett..

[13] Park C.-H., Chun K., Choi J.-H., Ji W.-K., Kim H.Y., Kim S.H., Han G., Kim M.-H. (2011). Synthesis and evaluation of tricyclic derivatives containing a non-aromatic amide as poly(ADP-ribose)polymerase-1 (PARP-1) inhibitors. Bull. Korean Chem. Soc..

[14] Park C.-H., Chun K., Joe B.-Y., Park J.-S., Kim Y.-C., Choi J.-S., Ryu D.-K., Koh S.-H., Cho G.W., Kim S.H. (2010). Synthesis and evaluation of tricyclic derivatives containing a non-aromatic amide as inhibitors of poly(ADP-ribose)polymerase-1 (PARP-1). Bioorg. Med. Chem. Lett..

[15] Lehtioe L., Jemth A.-S., Collins R., Loseva O., Johansson A., Markova N., Hammarstroem M., Flores A., Holmberg-Schiavone L., Weigelt J. (2009). Structural basis for inhibitor specificity in human poly(ADP-ribose)polymerase-3. J. Med. Chem..

[16] Tintori C., La Sala G., Vignaroli G., Botta L., Fallacara A.L., Falchi F., Radi M., Zamperini C., Dreassi E., Iacono L.D. (2015). Studies on the ATP binding site of Fyn kinase for the identification of new inhibitors and their evaluation as potential agents against tauopathies and tumors. J. Med. Chem..

[17] Brnardic E.J., Garbaccio R.M., Fraley M.E., Tasber E.S., Steen J.T., Arrington K.L., Dudkin V.Y., Hartman G.D., Stirdivant S.M., Drakas B.A. (2007). Optimization of a pyrazoloquinolinone class of Chk1 kinase inhibitors. Bioorg. Med. Chem. Lett..

[18] Huang S., Garbaccio R.M., Fraley M.E., Steen J., Kreatsoulas C., Hartman G., Stirdivant S., Drakas B., Rickert K., Walsh E. (2006). Development of 6-substituted indolylquinolinones as potent Chek1 kinase inhibitors. Bioorg. Med. Chem. Lett..

[19] Ni Z.-J., Barsanti P., Brammeier N., Diebes A., Poon D.J., Ng S., Pecchi S., Pfister K., Renhowe P.A., Ramurthy S. (2006). 4-(Aminoalkylamino)-3-benzimidazole-quinolinones as potent CHK-1 inhibitors. Bioorg. Med. Chem. Lett..

[20] Mugnaini C., Kostrzewa M., Bryk M., Mahmoud A.M., Brizzi A., Lamponi S., Giorgi G., Ferlenghi F., Vacondio F., Maccioni P. (2020). Design, synthesis, and physicochemical and pharmacological profiling of 7-hydroxy-5-oxopyrazolo[4,3-b]pyridine-6-carboxamide derivatives with antiosteoarthritic activity In Vivo. J. Med. Chem..

[21] Reyes-Batlle M., Freijo M.B., López-Arencibia A., Lorenzo-Morales J., McNaughton-Smith G., Pinero J.E., Abad-Grillo T. (2020). Identification of *N*-acyl quinolin-2(1*H*)-ones as new selective agents against clinical isolates of *Acanthamoeba* keratitis. Bioorg. Chem..

[22] Xue W., Li X., Ma G., Zhang H., Chen Y., Kirchmair J., Xia J., Wu S. (2020). *N*-Thiadiazole-4-hydroxy-2-quinolone-3-carboxamides bearing heteroaromatic rings as novel antibacterial agents: Design, synthesis, biological evaluation and target identification. Eur. J. Med. Chem..

[23] Yukawa T., Nakahata T., Okamoto R., Ishichi Y., Miyamoto Y., Nishimura S., Oikawa T., Kubo K., Adachi R., Satomi Y. (2020). Discovery of 1,8-naphthyridin-2-one derivative as a potent and selective sphingomyelin synthase 2 inhibitor. Bioorg. Med. Chem..

[24] Sekgota K.C., Majumder S., Isaacs M., Mnkandhla D., Hoppe H.C., Khanye S.D., Kriel F.H., Coates J., Kaye P.T. (2017). Application of the Morita-Baylis-Hillman reaction in the synthesis of 3-[(*N*-cycloalkylbenzamido)methyl]-2-quinolones as potential HIV-1 integrase inhibitors. Bioorg. Chem..

[25] Badaoui M.I., Magid A.A., Benkhaled M., Bensouici C., Harakat D., Voutquenne-Nazabadioko L., Haba H. (2019). Pyrroloquinolone A, a new alkaloid and other phytochemicals from *Atractylis cancellata* L. with antioxidant and anticholinesterase activities. Nat. Prod. Res..

[26] Lou L.-L., Cheng Z.-Y., Guo R., Yao G.-D., Song S.-J. (2019). Alkaloids from Juglans Mandshurica maxim induce distinctive cell death in hepatocellular carcinoma cells. Nat. Prod. Res..

[27] Choi Y.-H., Seo C., Jeong W., Lee J.E., Lee J.Y., Ahn E.-K., Kang J.-S., Lee J.-H., Choi C.W., Oh J.S. (2019). Glycopentanolones A-D, four new geranylated quinolone alkaloids from *Glycosmis pentaphylla*. Bioorg. Chem..

[28] Li X., Huo X., Li J., She X., Pan X. (2009). A concise synthesis of (±)-yaequinolone A2. Chin. J. Chem..

[29] Stephen A., Boyd D.R., Sharma N.D., Loke P.L. (2009). Syntheses and absolute configuration assignments of mono- and di-substituted chiral quinoline alkaloids obtained by asymmetric oxidation. Heterocycles.

[30] Chung H.S., Woo W.S. (2001). A quinolone alkaloid with antioxidant activity from the aleurone layer of anthocyanin-pigmented rice. J. Nat. Prod..

[31] Jacquemond-Collet I., Hannedouche S., Fourasté I., Moulis C. (2000). Novel quinoline alkaloid from trunk bark of *Galipea officinalis*. Fitoterapia.

[32] Akhmedzhanova V.I. (1999). New alkaloids from *Haplophyllum*. Chem. Nat. Compd..

[33] Chen I.-S., Wu S.-J., Leu Y.-L., Tsai I.-W., Wu T.-S. (1996). Alkaloids from root bark of *Zanthoxylum simulans*. Phytochemistry.

[34] Perrett S., Whitfield P.J. (1995). Atanine (3-dimethylallyl-4-methoxy-2-quinolone), an alkaloid with anthelmintic activity from the Chinese medicinal plant, *Evodia rutaecarpa*. Planta Med..

[35] Sarker S.D., Waterman P.G., Armstrong J.A. (1995). 3,4,8-Trimethoxy-2-quinolone: A new alkaloid from *Eriostemon gardneri*. J. Nat. Prod..

[36] Chen I.-S., Wu S.-J., Lin Y.-C., Tsai I.-L., Seki H., Ko F.-N., Teng C.-M. (1994). Dimeric 2-quinolone alkaloid and antiplatelet aggregation constituents of *Zanthoxylum simulans*. Phytochemistry.

[37] Wu S.-J., Chen I.-S. (1993). Alkaloids from *Zanthoxylum simulans*. Phytochemistry.

[38] Ngadjui B.T., Ayafor J.F., Mitaku S., Skaltsounis A.-L., Tillequin F., Koch M. (1989). Dehydration of hydroxy-hemiterpenoid quinoline alkaloids and synthesis of *Paraensidimerines*. J. Nat. Prod..

[39] Ayafor J.F., Sondengam B.L., Ngadjui B.T. (1982). Veprisilone, a prenylated 2-quinolone, and limonin from *Vepris louisii*. Phytochemistry.

[40] Vaquette J., Hifnawy M.S., Pousset J.L., Fournet A., Bouquet A., Cavé A. (1976). Alcaloides *d’Araliopsis soyauxii*. isolement d’un nouvel alcaloide, l’araliopsine. Phytochemistry.

[41] Rapoport H., Holden K.G. (1960). Alkaloids of *Balfourodendron riedelianum*. Balfourodine and isobalfourodine. J. Am. Chem. Soc..

[42] Rapoport H., Holden K.G. (1959). Isolation of alkaloids from *Balfourodendron riedelianum*. The Structure of balfourodine. J. Am. Chem. Soc..

[43] Goodwin S., Shoolery J.N., Horning E.C. (1959). Alkaloids of *Lunasia amara* Blanco. Hydroxylunacridine. J. Am. Chem. Soc..

[44] Hu Z., Deng Q., Yang S., Guo D. (2020). Preparation and fluorescence properties of novel 2-quinolone derivatives and their corresponding Eu(III) complexes. Colloids Surf. A.

[45] Lee S.B., Lee N.-G., Jung Y.R., Kim D., Hong K.B., Choi S. (2018). Synthesis and verification of fluorescent pH probes based on 2-quinolone platform. Chem. Lett..

[46] Murugan N.A.S., Yamesh V.U., Annaraj J. (2017). A Schiff’s base receptor for red fluorescence live cell imaging of Zn^2+^ ions in zebrafish embryos and naked eye detection of Ni^2+^ ions for bio-analytical applications. J. Mater. Chem. B.

[47] Velmurugan K., Raman A., Don D., Tang L., Easwaramoorthi S., Nandhakumar R. (2015). Quinoline benzimidazole-conjugate for the highly selective detection of Zn(II) by dual colorimetric and fluorescent turn-on responses. RSC Adv..

[48] Patil S.R., Nandre J.P., Patil P.A., Bothra S., Sahoo S.K., Klasek A., Rodríguez-López J., Mahulikar P.P., Patil U.D. (2014). Quinolone based chemosensor for the naked-eye and spectrophotometric detection of Cu^2+^ in aqueous media. Inorg. Chem. Commun..

[49] Ganesan P., Chandiran A., Gao P., Rajalingam R., Grätzel M., Nazeeruddin M.K. (2014). Molecular engineering of 2-quinolinone based anchoring groups for dye-sensitized solar cells. J. Phys. Chem. C.

[50] Murugavel S., Sundramoorthy S., Lakshmanan D., Subashini R., Kumar P.P. (2017). Synthesis, crystal structure analysis, spectral (NMR, FT-IR, FT-Raman and UV-Vis) investigations, molecular docking studies, antimicrobial studies and quantum chemical calculations of a novel 4-chloro-8-methoxyquinoline-2(1*H*)-one: An effective antimicrobial agent and an inhibition of DNA gyrase and lanosterol-14α-demethylase enzymes. J. Mol. Struct..

[51] Kieffer C., Cohen A., Verhaeghe P., Hutter S., Castera-Ducros C., Laget M., Remusat V., M’Rabet M.K., Rault S., Rathelot P. (2015). Looking for new antileishmanial derivatives in 8-nitroquinolin-2(1*H*)-one series. Eur. J. Med. Chem..

[52] Tashima T. (2015). The structural use of carbostyril in physiologically active substances. Bioorg. Med. Chem. Lett..

[53] Pedron J., Boudot C., Hutter S., Bourgeade-Delmas S., Stigliani J.-L., Sournia-Saquet A., Moreau A., Boutet-Robinet E., Paloque L., Mothes E. (2018). Novel 8-nitroquinolin-2(1*H*)-ones as NRT-bioactivated antiknetoplastid molecules: Synthesis, electrochemical and SAR study. Eur. J. Med. Chem..

[54] Kay I.T., Taylor P.J. (1968). The synthesis and physical properties of some 2-alkylthio-4-oxoquinoline-3-carboxylate esters. J. Chem. Soc. C.

[55] De la Cruz A., Elguero J., Goya P., Martínez A., Pfleiderer W. (1992). Tautomerism and acidity in 4-quinolone-3-carboxylic acid derivatives. Tetrahedron.

[56] Murguly E., Norsten T.B., Branda N. (1999). Tautomerism of 4-hydroxyterpyridine in the solid, solution and gas phase: An X-ray, FT-IR and NMR study. J. Chem. Soc. Perkin Trans. 2.

[57] Mphahlele M.J., El-Naha A.M. (2004). Tautomeric 2-arylquinolin-4(1*H*)-one derivatives- spectroscopic, X-ray and quantum chemical structural studies. J. Mol. Struct..

[58] Horta P., Kus N., Henriques M.S.C., Paixão J., Coelho L., Nogueira F., O’Neill P.M., Fausto R., Cristiano M.L.S. (2015). Quinolone-hydroxyquinoline tautomerism in quinolone 3-esters. Preserving the 4-oxoquinoline structure to retain antimalarial activity. J. Org. Chem..

[59] Kang O.-Y., Park S.J., Ahn H., Jeong K.C., Lim W.J. (2019). Structural assignment of the enol-keto tautomers of one-pot synthesized 4-hydroxyquinolines/4-quinolones. Org. Chem. Front..

[60] Ewing G.W., Steck E.A. (1946). Absorption spectra of heterocyclic compounds. I. quinolinols and isoquinolinols. J. Am. Chem. Soc..

[61] Pfister-Guillouzo G., Cuimon C., Frank J., Ellison J., Katritzky A.R. (1981). A photoelectron spectral study of the vapour phase tautomerism of 2- and 4-quinolone. Liebigs Ann. Chem..

[62] Mirek J., Sygula A. (1982). Semiempirical MNDO and UV absorption studies on tautomerism of 2-quinolones. Z. Für Naturforschung A.

[63] Masoud M.S., Mohammed M.S., Abdel-Latif F.F., Soliman E.M.A. (1988). Spectral studies on some 2-quinolones. Spectrosc. Lett..

[64] Pan Y., Lau K.-C., Al-Mogren M.M., Mahjoub A., Hochlaf M. (2014). Theoretical studies of 2-quinolinol: Geometries, vibrational frequencies, isomerization, tautomerism, and excited states. Chem. Phys. Lett..

[65] Lewis F.D., Reddy G.D., Elbert J.E., Tillberg B.E., Meltzer J.A., Kojima M. (1991). Spectroscopy and photochemistry of 2-quinolones and their Lewis acid complexes. J. Org. Chem..

[66] Krebs C., Förster W., Weiss C., Hofmann H.-J. (1982). Theoretical description of solvent effects. V. The medium influence on the lactim-lactam tautomerism of hydroxyazines. J. Prakt. Chem..

[67] Volle J.-N., Mävers U., Schlosser M. (2008). The tautomeric persistence of electronically and sterically biased 2-quinolones. Eur. J. Org. Chem..

[68] Bird C.W. (1986). The application of a new aromaticity index to six-membered ring heterocycles. Tetrahedron.

[69] Friedländer P. (1882). Ueber *o*-amidobenzaldehyd. Chem. Ber..

[70] Friedländer P., Gohring C.F. (1883). Ueber eine darstellungsmethode im pyridinkern substituirter Chinolinderivate. Chem. Ber..

[71] Knorr L. (1884). Synthesis of quinoline-derivatives. Liebigs Ann. Chem..

[72] Knorr L. (1886). Synthetical experiments with ethyl acetoacetate. Liebigs Ann. Chem..

[73] Knorr L. (1888). Syntheses with ethyl acetoacetate. Liebigs Ann. Chem..

[74] Bergstrom F.W. (1944). Heterocyclic nitrogen compounds. Part IIA. Hexacyclic compounds: Pyridine, quinoline, and isoquinoline. Chem. Rev..

[75] Hauser C.R., Reynolds G.A. (1948). Reactions of β-keto esters with aromatic amines. Syntheses of 2- and 4-hydroxyquinoline derivatives. J. Am. Chem. Soc..

[76] Staskun B. (1964). The conversion of benzoylacetanilides into 2- and 4-hydroxyquinolines. J. Org. Chem..

[77] Kumar K., Sai S., Gilbert T.M., Klumpp D.A. (2007). Knorr cyclizations and distonic superelectrophiles. J. Org. Chem..

[78] Abdou M.M. (2017). Chemistry of 4-hydroxy-2(1*H*)-quinolone. Part 1: Synthesis and reactions. Arab. J. Chem..

[79] Aly A.A., El-Sheref E.M., Mourad A.-F., Bakheet M.E., Bräse S. (2020). 4-Hydroxy-2-quinolones: Syntheses, reactions and fused heterocycles. Mol. Divers..

[80] Yamamoto Y. (2019). Recent advances in transition-metal-catalyzed synthesis of 3- and/or 4-aryl-2(1*H*)-quinolones. Heterocycles.

[81] Vessally E., Babazadeh M., Didehban K., Hosseinian A., Edjlali L. (2017). Intramolecular cyclization of *N*-arylpropiolamides: A new strategy for the synthesis of functionalized 2-quinolones. Curr. Org. Chem..

[82] Nishiwaki N. (2010). Chemistry of nitroquinolones and synthetic application to unnatural 1-methyl-2-quinolone derivatives. Molecules.

[83] Silva V.L.M., Silva A.M.S. (2019). Palladium-catalysed synthesis and transformation of quinolones. Molecules.

[84] Cortese N.A., Ziegler C.B., Hrnjez B.J., Heck R.F. (1978). Palladium-catalyzed synthesis of 2-quinolone derivatives from 2-iodoanilines. J. Org. Chem..

[85] Bernini R., Cacchi S., Fabrizi G., Sferrazza A. (2006). 4-Aryl-2-quinolones via a domino Heck reaction/cyclization process. Heterocycles.

[86] Battistuzzi G., Bernini R., Cacchi S., De Salve I., Fabrizi G. (2007). 4-Aryl-2-quinolones through a pseudo-domino Heck/Buchwald–Hartwig reaction in a molten tetrabutylammonium acetate/tetrabutylammonium bromide mixture. Adv. Synth. Catal..

[87] Al-Amin M., Honma T., Hoshiya N., Shuto S., Arisawa M. (2012). Ligand-free Buchwald–Hartwig aromatic aminations of aryl halides catalyzed by low-leaching and highly recyclable sulfur-modified gold-supported palladium material. Adv. Synth. Catal..

[88] Cho C.S., Kim J.U. (2007). An approach for quinolines via palladium-catalyzed Heck coupling followed by cyclization. Tetrahedron Lett..

[89] Borhade S.R., Waghmode S.B. (2011). An efficient synthesis of 4-arylquinolin-2(1*H*)-ones and 3-alkenyl-4-arylquinolin-2(1*H*)-one using a Pd/NiFe_2_O_4_-catalyzed consecutive Heck reaction. Can. J. Chem..

[90] Manley P.J., Bilodeau M.T. (2004). A new synthesis of naphthyridinones and quinolinones: Palladium-catalyzed amidation of *o*-carbonyl-substituted aryl halides. Org. Lett..

[91] Teng Y., Suwanarusk R., Ngai M.H., Srinivasan R., Ong A.S.M., Ho B., Rénia L., Chai C.L.L. (2015). An amidation/cyclization approach to the synthesis of *N*-hydroxyquinolinones and their biological evaluation as potential anti-plasmodial, anti-bacterial and iron(II)-chelating agents. Bioorg. Med. Chem. Lett..

[92] Dong Y., Liu B., Chen P., Liu Q., Wang M. (2014). Palladium-catalyzed C–S activation/aryne insertion/coupling sequence: Synthesis of functionalized 2-quinolinones. Angew. Chem. Int. Ed..

[93] Wang W., Peng X., Qin X., Zhao X., Ma C., Tung C.-H., Xu Z. (2015). Synthesis of quinolinones with palladium-catalyzed oxidative annulation between acrylamides and arynes. J. Org. Chem..

[94] Wu J., Xiang S., Zeng J., Leow M., Liu X.-W. (2015). Practical route to 2-quinolinones via a Pd-catalyzed C–H bond activation/C–C bond formation/cyclization cascade reaction. Org. Lett..

[95] Xiao C., Wang Z., Lei M., Hu L. (2017). Pd(II)-catalyzed cascade Heck/intramolecular C(*sp*^2^)–H amidation reaction: An efficient route to 4-aryl-2-quinolinones. Tetrahedron.

[96] Fu L., Huang X., Wang D., Zhao P., Ding K. (2011). Copper(I) iodide catalyzed synthesis of quinolinones via cascade reactions of 2-halobenzocarbonyls with 2-arylacetamides. Synthesis.

[97] Ahn B.H., Lee I.Y., Lim H.N. (2018). Step-economical synthesis of 3-amido-2-quinolones by dendritic copper powder-mediated one-pot reaction. Org. Biomol. Chem..

[98] Mai W.-P., Sun G.-C., Wang J.-T., Song G., Mao P., Yang L.-R., Yuan J.-W., Xiao Y.-M., Qu L.-B. (2014). Silver-catalyzed radical tandem cyclization: An approach to direct synthesis of 3-acyl-4-arylquinolin-2(1*H*)-ones. J. Org. Chem..

[99] Chen C., Hao Y., Zhang T.-Y., Pan J.-L., Ding J., Xiang H.-Y., Whang M., Ding T.-M., Duan A., Zhang S.-Y. (2019). Computational and experimental studies on copper-mediated selective cascade C–H/N–H annulation of electron-deficient acrylamide with arynes. Chem. Commun..

[100] Charoenpol A., Meesin J., Khaikate O., Reutrakul V., Pohmakotr M., Leowanawat P., Soorukram D., Kuhakarn C. (2018). Synthesis of 3-substituted quinolin-2(1*H*)-ones via the cyclization of *o*-alkynylisocyanobenzenes. Org. Biomol. Chem..

[101] Manikandan R., Jeganmohan M. (2014). Ruthenium-catalyzed cyclization of anilides with substituted propiolates or acrylates: An efficient route to 2-quinolones. Org. Lett..

[102] Zhu Y.-Q., Hui L.-W., Niu Y.-X., Lv L.-G., Zhu K. (2019). Reaction of cycloalkene-1-carboxamides with aryl boronates via rhodium(III)-catalyzed C–H activation: A versatile route to 3,4-cycloalkaquinolin-2(1*H*)-ones. Adv. Synth. Catal..

[103] Zeng R., Dong G. (2015). Rh-catalyzed decarbonylative coupling with alkynes via C–C bond activation of isatins. J. Am. Chem. Soc..

[104] Jia C.-S., Dong Y.-W., Tu S.-J., Wang G.-W. (2007). Microwave-assisted solvent-free synthesis of substituted 2-quinolones. Tetrahedron.

[105] Iwai T., Fujihara T., Terao J., Tsuji Y. (2010). Iridium-catalyzed annulation of *N*-arylcarbamoyl chlorides with internal alkynes. J. Am. Chem. Soc..

[106] Wang Y.-X., Zhang F.-P., Luan Y.-X., Ye M. (2020). Ligand-enabled Ni−Al bimetallic catalysis for nonchelated dual C−H annulation of arylformamides and alkynes. Org. Lett..

[107] Domínguez-Fernández F., López-Sanz J., Pérez-Mayoral E., Bek D., Martín-Aranda R.M., López-Peinado A.J., Čejka J. (2009). Novel basic mesoporous catalysts for the Friedländer reaction from 2-aminoaryl ketones: Quinolin-2(1*H*)-ones versus quinolones. ChemCatChem.

[108] López-Sanz J., Pérez-Mayoral E., Procházková D., Martín-Aranda R.M., López-Peinado A.J. (2010). Zeolite promoting quinoline synthesis via Friedländer reaction. Top. Catal..

[109] Subashini R., Khan F.-R.N. (2012). Solvent-free synthesis and antibacterial studies of some quinolinones. Monatsh. Chem..

[110] Bui C.T. (2014). One-pot microwave-assisted synthesis of 3,4-disubstituted 2-quinolinones. Synth. Commun..

[111] Huang Y.-N., Li Y.-L., Li J., Deng J. (2016). Beyond a protecting reagent: DMAP-catalyzed cyclization of Boc-anhydride with 2-alkenylanilines. J. Org. Chem..

[112] Knölker H.-J., Braxmeier T., Schlechtingen G. (1995). A novel method for the synthesis of isocyanates under mild conditions. Angew. Chem. Int. Ed..

[113] Aksenov A.V., Smirnov A.N., Aksenov N.A., Aksenova I.V., Frolova L.V., Kornienko A., Magedov I.V., Rubin M. (2013). Metal-free transannulation reaction of indoles with nitrostyrenes: A simple practical synthesis of 3-substituted 2-quinolones. Chem. Commun..

[114] Aksenov A.V., Smirnov A.N., Aksenov N.A., Aksenova I.V., Bijieva A.S., Rubin M. (2014). Highly efficient modular metal-free synthesis of 3-substituted 2-quinolones. Org. Biomol. Chem..

[115] Aksenov A.V., Smirnov A.N., Aksenov N.A., Aksenova I.V., Matheny J.P., Rubin M. (2015). Metal-free ring expansion of indoles with nitroalkenes: A simple, modular approach to 3-substituted 2-quinolones. RSC Adv..

[116] Zhang W.-Z., Liu S., Lu X.-B. (2015). DBU-promoted carboxylative cyclization of *o*-hydroxy- and *o*-acetamidoacetophenone. Beilstein J. Org. Chem..

[117] Zhang Z., Liao L.-L., Yan S.-S., Wang L., He Y.-Q., Ye J.-H., Li J., Zhi Y.-G., Yu D.-G. (2016). Lactamization of sp^2^ C–H bonds with CO_2_: Transition-metal-free and redox-neutral. Angew. Chem. Int. Ed..

[118] Kadnikov D.V., Larock R.C. (2003). Palladium-catalyzed carbonylative annulation of terminal alkynes: Synthesis of coumarins and 2-quinolones. J. Organomet. Chem..

[119] Kadnikov D.V., Larock R.C. (2004). Synthesis of 2-quinolones via palladium-catalyzed carbonylative annulation of internal alkynes by *N*-substituted *o*-iodoanilines. J. Org. Chem..

[120] Chen J., Natte K., Spannenberg A., Neumann H., Beller M., Wu X.-F. (2014). Palladium-catalyzed carbonylative [3+2+1] annulation of *N*-aryl-pyridine-2-amines with internal alkynes by C–H activation: Facile synthesis of 2-quinolinones. Chem. Eur. J..

[121] Jafarpour F., Otaredi-Kashani A. (2014). Carbonylative annulation of unsaturated compounds using molybdenum hexacarbonyl: An efficient synthesis of 2(1*H*)-quinolones. ARKIVOC.

[122] Chen J.-R., Liao J., Xiao W.-J. (2010). Microwave-assisted, palladium-catalyzed carbonylative cyclization—Rapid synthesis of 2-quinolones from unprotected 2-iodoanilines and terminal alkynes. Can. J. Chem..

[123] Thakur V., Sharma A., Yamini, Sharma N., Das P. (2019). Supported palladium nanoparticles-catalyzed synthesis of 3-substituted 2-quinolones from 2-iodoanilines and alkynes using oxalic acid as C1 source. Adv. Synth. Catal..

[124] Tadd A.C., Matsuno A., Fielding M.R., Willis M.C. (2009). Cascade palladium-catalyzed alkenyl aminocarbonylation/intramolecular aryl amidation: An annulative synthesis of 2-quinolones. Org. Lett..

[125] Sun S., Hu W.-M., Gu N., Cheng J. (2016). Palladium-catalyzed multi-component reactions of *N*-tosylhydrazones, 2-iodoanilines and CO_2_ towards 4-aryl-2-quinolinones. Chem. Eur. J..

[126] Li X., Li X., Jiao N. (2015). Rh-catalyzed construction of quinolin-2(1*H*)-ones via C–H bond activation of simple anilines with CO and alkynes. J. Am. Chem. Soc..

[127] Li X., Pan J., Wu H., Jiao N. (2017). Rh-catalyzed aerobic oxidative cyclization of aniline, alkynes, and CO. Chem. Sci..

[128] Zhu F., Li Y., Wang Z., Wu X.-F. (2016). Iridium-catalyzed carbonylative synthesis of halogen-containing quinoline-2(1*H*)-ones from internal alkynes and simple anilines. Adv. Synth. Catal..

[129] Kim A.R., Lim H.N. (2020). One-pot copper-catalyzed three-component reaction: A modular approach to functionalized 2-quinolones. RSC. Adv..

[130] Marcaccini S., Pepino R., Pozo M.C., Basurto S., García-Valverde M., Torroba T. (2004). One-pot synthesis of quinolin-2-(1*H*)-ones via tandem Ugi-Knoevenagel condensations. Tetrahedron Lett..

[131] Kaïm L.E., Grimaud L., Oble J. (2005). Phenol Ugi-Smiles systems: Strategies for the multicomponent *N*-arylation of primary amines with isocyanides, aldehydes, and phenols. Angew. Chem. Int. Ed..

[132] Heiden G.V., Jong J.A.W., Ruijter E., Orru R.V.A. (2016). 2-Bromo-6-isocyanopyridine as a universal convertible isocyanide for multicomponent chemistry. Org. Lett..

[133] Gordon C.P., Young K.A., Hizartzidis L.H., Deane F.M., McCluskey A. (2011). Investigation of the one-pot synthesis of quinolin-2-(1*H*)-ones and the discovery of a variation of the three-component Ugi reaction. Org. Biomol. Chem..

[134] Rasouli M.A., Mahdavi M., Saeedi M., Ranjbar P.R., Shafiee A., Foroumadi A. (2015). Synthesis of novel 2-oxoquinoline derivatives via Ugi-four-component-Heck reaction. J. Heterocycl. Chem..

[135] Du X., Huang J., Nechaev A.A., Yao R., Gong J., Eycken E.V.V., Pereshivko O.P., Peshkov V.A. (2018). Gold-catalyzed post-Ugi alkyne hydroarylation for the synthesis of 2-quinolones. Beilstein J. Org. Chem..

[136] Singh K., Malviya B.K., Verma V.P., Badsara S.S., Bhardwaj V.K., Sharma S. (2019). Cationic Pd(II) catalyzed regioselective intramolecular hydroarylation for the efficient synthesis of 4-aryl-2-quinolones. Tetrahedron.

[137] Wang Q., Osipyan A., Konstantinidou M., Butera R., Mgimpatsang K.C., Shishkina S., Dömling A. (2019). Pd-catalyzed *de novo* assembly of diversely substituted indole-fused polyheterocycles. J. Org. Chem..

[138] Zhang T.-Y., Liu C., Chen C., Liu J.-X., Xiang H.-Y., Jiang W., Ding T.-M., Zhang S.-Y. (2018). Copper-mediated cascade C–H/N–H annulation of indolocarboxamides with arynes: Construction of tetracyclic indoloquinoline alkaloids. Org. Lett..

[139] Ding D., Zhu G., Jiang X. (2018). Ligand-controlled palladium(II)-catalyzed regiodivergent carbonylation of alkynes: Syntheses of indolo[3,2-c]coumarins and benzofuro[3,2-c]quinolinones. Angew. Chem. Int. Ed..

